# Time- and Dose-Dependent PSP-Induced Modulation of Antiviral Signaling Networks in CD4+ T Cells

**DOI:** 10.3390/ijms27083661

**Published:** 2026-04-20

**Authors:** Glamaris N. Rosario-Sanfiorenzo, Giovanni O. Alicea-Pérez, Ashlin N. Álvarez-Flores, Naiara I. Hernández-Santisteban, Amanda C. Rivera-Payán, Jeshua J. Colón-Fernández, Abigail M. Rivera-Berganzo, Victoria Bermudez-Fosse, Ileanmarie Santana-Costas, Carolina Nieves-Moreno, Fabiola I. Colón-Santiago, Julieness M. Correa-Haifa, Natalia I. Sánchez-Otero, Geraldine Cintrón-Vélez, Génesis M. Matos-Morales, Eduardo Álvarez-Rivera

**Affiliations:** 1 Department of Science and Technology, Interamerican University of Puerto Rico, Metropolitan Campus, San Juan, PR 00926, USA; glamaris.nicolerosar@intermetro.edu (G.N.R.-S.); naiarai.hernandezsan@intermetro.edu (N.I.H.-S.); amandac.riverapayan@intermetro.edu (A.C.R.-P.); jeshuaj.colon@intermetro.edu (J.J.C.-F.); genesis.matosmorales@intermetro.edu (G.M.M.-M.); 2 Department of Biology, Universidad de Puerto Rico, Bayamón, PR 00960, USA; giovanni.alicea2@upr.edu (G.O.A.-P.); julieness.correa@upr.edu (J.M.C.-H.); 3 Department of Biology, Universidad de Puerto Rico, Arecibo, PR 00612, USA; ashlin.alvarez@upr.edu (A.N.Á.-F.); carolina.nieves3@upr.edu (C.N.-M.); 4 School of Medicine, Universidad Central del Caribe, Bayamón, PR 00960, USA; victoriaisabell444@icloud.com (V.B.-F.); 122fcolon@uccaribe.edu (F.I.C.-S.); 5 James L. Winkle College of Pharmacy, University of Cincinnati, Cincinnati, OH 45221, USA; 6 Department of Biology, Universidad de Puerto Rico, Río Piedras, PR 00925, USA; geraldine.cintron@upr.edu; 7 Department of Microbiology and Immunology, School of Medicine, Universidad Central del Caribe, Bayamón, PR 00960, USA

**Keywords:** polysaccharide peptide, *Coriolus versicolor*, Jurkat T cells, interferon, protein kinase R, cofilin-1, signal transducer and activator of transcription, nuclear factor kappa B, toll-like receptor 4

## Abstract

Natural bioactive polysaccharides have been investigated for their ability to modulate antiviral immune responses. Polysaccharide peptide (PSP) from *Coriolus versicolor* previously restricted human immunodeficiency virus type 1 (HIV-1) entry into monocytic cells through a protein kinase R (PKR)-dependent cytoskeletal mechanism. However, its impact on antiviral signaling in adaptive cluster of differentiation 4 (CD4)+ T-cell models remains incompletely defined. Here, we evaluated concentration- and time-dependent effects of PSP (50–1000 µg/mL) in Jurkat T cells over 3 and 6 days. Cell viability was assessed by MTT, trypan blue exclusion, and viable cell density analysis. Immunoblotting and reverse transcription quantitative polymerase chain reaction (RT-qPCR) were performed to examine Toll-like receptor 4 (TLR4), nuclear factor kappa B (NF-κB), signal transducer and activator of transcription 1 and 2 (STAT1/STAT2), PKR, interferon gamma (IFN-γ), and cofilin-1 signaling. PSP did not induce cytotoxicity at any concentration. Instead, PSP promoted dose- and time-dependent upregulation of intracellular TLR4, PKR, phospho-PKR (Thr446), Cofilin-1, phospho-Cofilin-1 (Ser3), phospho-STAT1 (Tyr701), phospho-STAT2 (Tyr690), phospho-NF-κB (Ser536), and IFN-γ, with amplified responses at Day 6. These changes were paralleled by transcriptional induction of antiviral-associated genes. Collectively, PSP induces coordinated interferon (IFN)-associated and cytoskeletal regulatory signaling in Jurkat T cells without cytotoxicity, providing a mechanistic framework for future evaluation of viral permissiveness and antiviral responses in adaptive immune models.

## 1. Introduction

Despite substantial advances in prevention strategies and antiretroviral therapy, human immunodeficiency virus type 1 (HIV-1) continues to pose a significant global public health challenge. Recent estimates indicate that approximately 40.8 million individuals were living with HIV worldwide at the end of 2024, with nearly 1.3 million new infections and approximately 630,000 AIDS-related deaths reported during the same year [1,2,3]. Although antiretroviral therapy has transformed HIV infection into a manageable chronic condition, it does not eradicate viral reservoirs nor fully prevent transmission, underscoring the need for complementary strategies that enhance host antiviral control and limit viral persistence [4,5].

**Figure 1:**
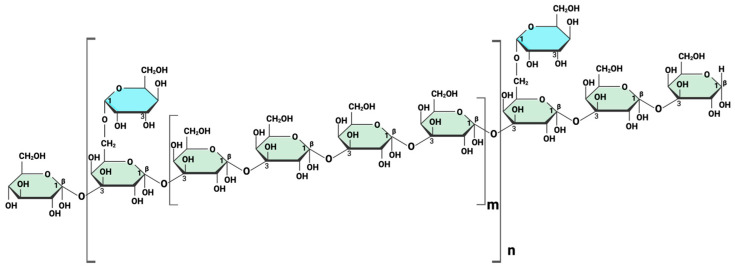
Representative schematic structure of polysaccharide peptide (PSP) illustrating its β-glucan backbone and branching features. PSP is composed of a β-(1→3)-linked glucan backbone (green) with β-(1→6)-linked branching units (blue). The bracketed regions indicate variable repeating units within the polymer, where n denotes the degree of polymerization of the backbone and m represents the extent of branching along the chain. This diagram provides a simplified structural representation and does not capture the full molecular heterogeneity of PSP. Created in BioRender. Alvarez-Rivera, E. (2026) https://BioRender.com/3mifup5 (accessed on 10 April 2026).

Natural bioactive compounds have attracted increasing attention as immunomodulatory agents that can influence antiviral signaling pathways [6]. Polysaccharide peptide (PSP), derived from the medicinal mushroom *Coriolus versicolor* (also known as Trametes versicolor), is a protein-bound polysaccharide extract widely used for its immune-supportive properties [7]. PSP has been reported to exert anti-inflammatory, antioxidant, antiviral-associated, and immunostimulatory effects in certain immune cell models [8,9]. PSP consists of a complex β-glucan backbone with branching polysaccharide structures characteristic of immunomodulatory fungal-derived compounds. A schematic representation of PSP’s structure is shown in Figure 1 to illustrate its general molecular architecture; however, this representation is simplified and does not capture the full structural heterogeneity of PSP [7].

From a mechanistic perspective, PSP has been shown to modulate innate immune signaling pathways, including Toll-like receptor (TLR)-associated cascades and interferon (IFN)-related responses [8]. Notably, our previous study demonstrated that PSP significantly restricted HIV-1 entry in THP-1 monocytic cells by approximately 73%, an effect associated with protein kinase R (PKR) activation and subsequent phosphorylation of Cofilin-1 at serine 3 [9]. This PKR-dependent modulation of cytoskeletal dynamics interfered with actin remodeling processes required for viral entry, identifying a host-directed antiviral regulatory mechanism.

HIV-1 infection is profoundly influenced by host signaling pathways that regulate antiviral gene expression and cytoskeletal organization. Toll-like receptor 4 (TLR4)-mediated signaling activates downstream transcriptional programs involving nuclear factor kappa B (NF-κB) and IFN-regulated pathways [9,10]. Signal transducer and activator of transcription (STAT) 1 and STAT2 phosphorylation play a central role in IFN-driven antiviral responses, while PKR functions as an IFN-stimulated kinase implicated in viral restriction and translational control [11,12]. Additionally, cofilin-mediated actin remodeling is essential for HIV-1 entry and intracellular trafficking in cluster of differentiation 4 (CD4)+ T cells [13,14]. HIV-1 actively manipulates these signaling axes to promote infection, persistence, and immune evasion [13].

While PSP has been reported to influence T-cell activity in mixed lymphocyte populations and peripheral blood mononuclear cell systems [8], systematic characterization of its concentration- and time-dependent effects on defined antiviral signaling nodes within CD4+ T-cell models remains limited. Given that CD4+ T lymphocytes are the principal cellular targets of HIV-1 infection and major viral reservoirs, determining whether PSP induces coordinated antiviral signaling programs in T-cell systems is a necessary mechanistic step before evaluating direct effects on viral permissiveness.

In the present study, we investigated whether PSP elicits concentration- and time-dependent modulation of key antiviral signaling nodes, including TLR4, NF-κB, STAT1, STAT2, PKR, interferon gamma (IFN-γ), and Cofilin-1, in Jurkat T cells, a widely used in vitro model of human CD4+ T lymphocytes. These pathways are well-established regulators of HIV-1 infection and persistence in CD4+ T cells [8,9]. Rather than directly assessing HIV-1 infection, this study was designed to first establish whether PSP induces coordinated immunoregulatory signaling in adaptive T cells without inducing cytotoxicity. By defining the molecular framework induced by PSP in Jurkat cells, this work provides a mechanistic framework for future studies investigating whether PSP-modulated signaling environments influence viral susceptibility in adaptive immune contexts.

## 2. Results

### 2.1. PSP Does Not Induce Cytotoxicity in Jurkat T Cells at Concentrations up to 1000 μg

**Figure 2:**
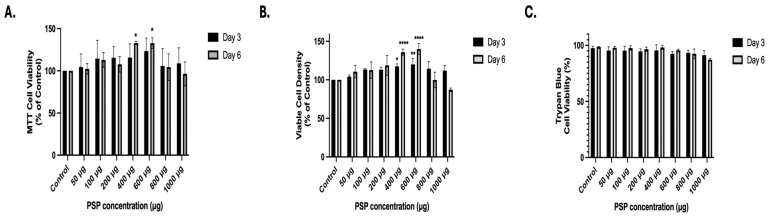
Effects of polysaccharide peptide (PSP) treatment on Jurkat T-cell viability. (**A**) Thiazolyl blue tetrazolium bromide (MTT) assay evaluating mitochondrial metabolic activity following PSP treatment (50–1000 µg/mL) for 3 and 6 days, with replenishment at day 3. (**B**) Quantification of viable cell density following PSP exposure, expressed as percentage of untreated control. (**C**) Trypan blue exclusion assay assessing membrane integrity and total cell viability after 6 days of treatment. For all experiments, Jurkat T cells were treated with PSP at the indicated concentrations beginning at day 0 and replenished at day 3. Total exposure time was 6 days. Viability and viable cell density were normalized to untreated controls. Data are presented as mean ± standard deviation (SD) from three independent biological replicates. Statistical significance was determined using two-way analysis of variance (ANOVA) followed by Dunnett’s multiple comparisons test (vs. corresponding control within each time point). * *p* ≤ 0.05, ** *p* ≤ 0.01, **** *p* ≤ 0.0001.

To determine whether PSP affects Jurkat T-cell viability, cells were treated with increasing concentrations of PSP (50–1000 µg/mL) for 3 or 6 days. RPMI-1640 media containing fresh PSP were replenished every 3 days. Cell viability and growth were evaluated using three complementary approaches: Thiazolyl blue tetrazolium bromide (MTT) metabolic activity assays (Figure 2A), viable cell density determination (Figure 2B), and trypan blue exclusion assays (Figure 2C).

MTT analysis revealed that PSP treatment did not significantly reduce metabolic activity at any concentration tested at either time point (Figure 2A). Instead, a modest increase in MTT signal was observed at intermediate concentrations (400–600 µg), particularly at Day 6. Statistical analysis using two-way analysis of variance (ANOVA) followed by Dunnett’s multiple comparison test indicated significant increases relative to controls at selected concentrations of 400 μg and 600 μg during a 6-day period, while higher concentrations (800–1000 µg) did not produce a reduction below control levels. These findings indicate that PSP does not impair mitochondrial metabolic activity in Jurkat T cells under the experimental conditions tested.

To further assess cell recovery, viable cell density was quantified by trypan blue exclusion and normalized to the control (Figure 2B). Consistent with the MTT results, PSP did not reduce viable cell numbers at any concentration or time point. A significant increase in viable cell density was observed at intermediate concentrations (400–600 μg) at Day 3 and 6, indicating that PSP does not exert cytotoxic or cytostatic effects within this range. Notably, no reduction in viable cell density was detected at the highest concentration tested (1000 µg/mL).

To directly assess cell membrane integrity, the percentage of viable cells was determined by trypan blue staining (Figure 2C). Across all concentrations and time points, cell viability remained consistently high (±87–99%), with no significant reduction relative to controls. These data confirm that PSP treatment does not compromise membrane integrity or induce overt cytotoxicity in Jurkat T cells.

Collectively, these results demonstrate that PSP, at concentrations up to 1000 µg and under repeated exposure conditions for up to 6 days, does not induce cytotoxicity in Jurkat T cells. The absence of reductions in metabolic activity, viable cell density, or membrane integrity establishes a non-toxic concentration range suitable for subsequent mechanistic analyses.

### 2.2. PSP Modulates Innate and Antiviral Signaling Pathways in Jurkat T Cells in a Concentration- and Time-Dependent Manner

To determine whether PSP modulates antiviral-associated signaling pathways in adaptive immune cells, Jurkat T cells were treated with increasing concentrations of PSP (50–1000 µg/mL) for 3 or 6 days in RPMI 1640 media, with the media and compound refreshed every 72 h. Protein expression was assessed by Western blot for whole-cell lysates.

#### 2.2.1. Upregulation of TLR4 and PKR Signaling Components

**Figure 3:**
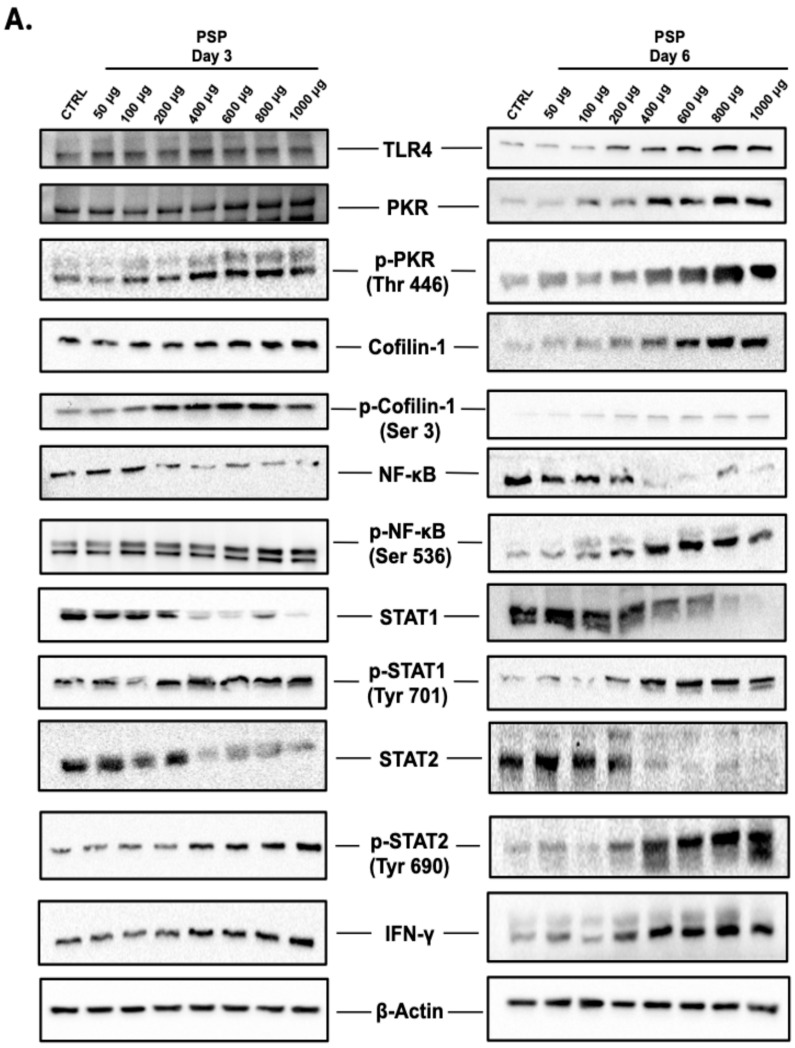

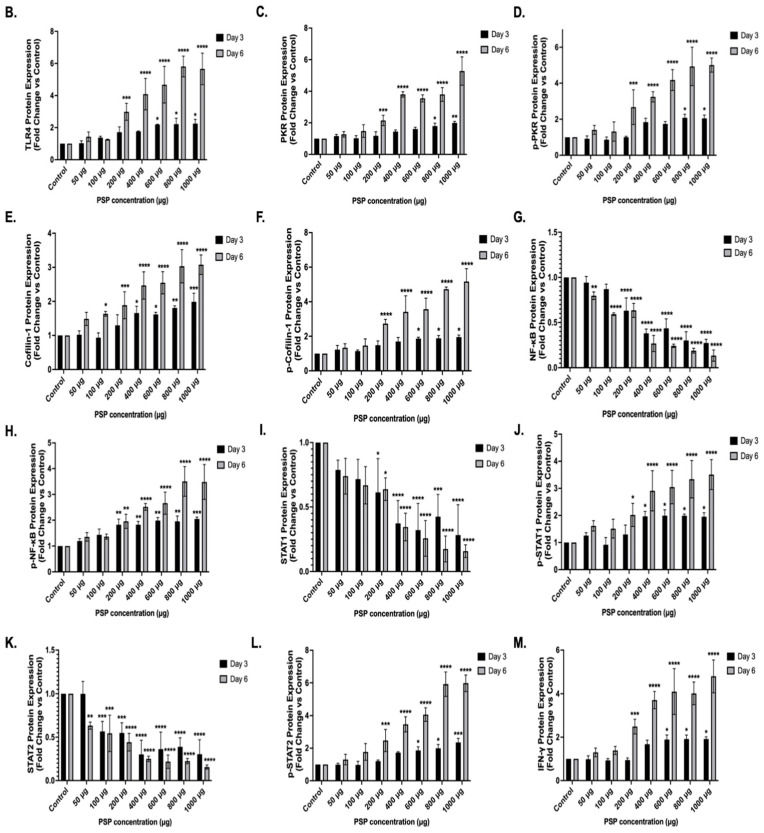
Concentration- and time-dependent modulation of anti-viral signaling and IFN-related proteins in polysaccharide peptide (PSP)-treated Jurkat T cells. (**A**) Representative Western blot images. (**B**–**M**) Quantitative immunoblot analysis of total cellular protein expression levels following PSP treatment (50–1000 µg/mL) for 3 or 6 days. Proteins analyzed include (**B**) Toll-like receptor 4 (TLR4), (**C**) Protein Kinase R (PKR), (**D**) phospho-PKR (Thr446), (**E**) Cofilin-1, (**F**) phospho-Cofilin-1 (Ser3), (**G**) Nuclear factor kappa B (NF-κB), (**H**) phospho-NF-κB (Ser536), (**I**) Signal transducer and activator of transcription (STAT) 1, (**J**) phospho-STAT1 (Tyr701), (**K**) STAT2, (**L**) phospho-STAT2 (Tyr690), and (**M**) intracellular interferon gamma (IFN-γ). β-Actin was used as a loading control and for normalization of densitometric values. Band intensities were quantified using ImageJ software (National Institutes of Health, Bethesda, MD, USA, version 1.54g) by measuring integrated density and normalizing to the corresponding β-actin signal. Data are expressed as fold change relative to untreated control and presented as mean ± standard deviation (SD) (n = 3 independent biological replicates). Statistical significance was determined using two-way analysis of variance (ANOVA) followed by Dunnett’s multiple comparisons test (vs. corresponding control within each time point). * *p* ≤ 0.05, ** *p* ≤ 0.01, *** *p* ≤ 0.001, **** *p* ≤ 0.0001. Representative full-length, original, and uncropped immunoblots are provided in Supplementary Figure S1.

PSP treatment resulted in a concentration- and time-dependent increase in total cellular TLR4 protein expression, as assessed by immunoblotting of whole-cell lysates (Figure 3B). At Day 3, statistically significant induction was first observed at 600 µg (2.18-fold of the control) and remained elevated through 1000 µg (2.24-fold of the control). At Day 6, significant increases were detected at lower concentrations, beginning at 200 µg (2.98-fold of the control) and progressively rising to 5.66-fold at 1000 µg. Statistical analysis revealed significant main effects of concentration and treatment duration, with a significant interaction indicating enhanced induction over prolonged exposure.

Similarly, total PKR protein expression demonstrated concentration-dependent upregulation (Figure 3C). At Day 3, significant increases were observed beginning at 800 µg (1.79-fold of the control) and reached 1.99-fold at 1000 µg. At Day 6, induction was detected at lower concentrations, starting at 200 µg (2.14-fold of the control) and increasing to 5.27-fold at 1000 µg.

Phosphorylated PKR at threonine 446 (p-PKR Thr446) also exhibited significant concentration- and time-dependent induction (Figure 3D). At Day 3, phosphorylation was significantly elevated at 800 µg (2.08-fold of the control) and remained elevated at 1000 µg (2.04-fold). At Day 6, p-PKR induction began at 200 µg (2.66-fold of the control) and increased to 5.00-fold at 1000 µg. Statistical analysis confirmed significant main effects of concentration and treatment duration, as well as a significant interaction between these variables.

Collectively, both total PKR and p-PKR (Thr446) demonstrated parallel upregulation, with more pronounced responses observed at Day 6.

#### 2.2.2. Modulation of Actin-Regulatory Proteins: Cofilin-1 and p-Cofilin-1

Similar to the previous findings, PSP treatment significantly increased total Cofilin-1 protein expression in a concentration- and time-dependent manner compared to untreated controls (Figure 3E). At Day 3, significant upregulation was observed, beginning at 400 µg (1.65-fold control) and increasing to 1.98-fold at 1000 µg. At Day 6, induction was detectable at lower concentrations, beginning at 100 µg (1.63-fold of the control) and progressively increasing to 3.07-fold at 1000 µg. Two-way ANOVA revealed significant main effects of concentration and treatment duration, with greater magnitude observed at Day 6.

Phosphorylated Cofilin-1 at serine 3 (p-Cofilin-1 Ser3) also demonstrated significant concentration- and time-dependent induction (Figure 3F). At Day 3, significant increases were detected at 600 µg (1.86-fold of the control) and remained elevated at 1000 µg (1.95-fold). At Day 6, phosphorylation was induced at lower concentrations, beginning at 200 µg (2.73-fold of the control) and increasing to 5.16-fold at 1000 µg. Statistical analysis confirmed significant effects of concentration and time, with a significant interaction between these variables.

Collectively, PSP treatment resulted in parallel increases in total Cofilin-1 and its Ser3-phosphorylated form, with enhanced induction observed at prolonged exposure.

#### 2.2.3. Differential Regulation of NFκB Signaling Components

Total NFκB (p65 subunit) protein expression demonstrated a concentration-dependent reduction following PSP treatment at both time points (Figure 3G). At Day 3, statistically significant decreases were observed beginning at 200 µg (0.63-fold relative to control), progressively declining to 0.27-fold at 1000 µg. At Day 6, suppression of total NFκB was detected at lower concentrations, with significance beginning at 50 µg (0.79-fold of the control) and further decreasing to 0.13-fold at 1000 µg.

In contrast, phosphorylation of NFκB at serine 536 (p-NFκB Ser536), corresponding to activation-associated modification of the p65 subunit, exhibited a concentration-dependent increase (Figure 3H). At Day 3, significant induction began at 200 µg (+1.82-fold relative to the control) and increased to +2.04-fold at 1000 µg. At Day 6, phosphorylation was similarly induced starting at 200 µg (+1.95-fold) and reached +3.48-fold at 1000 µg. Two-way ANOVA revealed significant main effects of concentration and treatment duration, with enhanced magnitude of phosphorylation observed at Day 6.

Thus, PSP treatment was associated with a reciprocal pattern characterized by reduced total NFκB (p65) abundance and increased phosphorylation at Ser536 in a concentration- and time-dependent manner.

#### 2.2.4. Reciprocal Regulation of STAT1 and STAT2 Signaling

Total STAT1 protein expression progressively decreased with increasing PSP concentration at both time points (Figure 3I). At Day 3, significant reductions were detected beginning at 200 µg (0.61-fold of the control) and declined further to 0.28-fold at 1000 µg. At Day 6, significant suppression was similarly observed starting at 200 µg (0.63-fold of the control), with expression reduced to 0.15-fold at 1000 µg.

In contrast, phosphorylation of STAT1 at tyrosine 701 (p-STAT1 Tyr701) was significantly increased following PSP treatment (Figure 3J). At Day 3, significant induction was observed at 400 µg (+1.96-fold of the control) and remained elevated through 1000 µg (+1.97-fold). At Day 6, phosphorylation was induced at lower concentrations, beginning at 200 µg (+2.01-fold) and increasing to +3.50-fold at 1000 µg. Two-way ANOVA revealed significant main effects of concentration and treatment duration, with a greater magnitude of phosphorylation at Day 6.

Total STAT2 expression exhibited an even more pronounced concentration-dependent reduction (Figure 3K). At Day 3, significant decreases were observed, beginning at 100 µg (0.56-fold control) and declining to 0.30-fold at 1000 µg. At Day 6, suppression was detectable starting at 50 µg (0.63-fold) and further reduced to 0.15-fold at 1000 µg.

Conversely, phosphorylation of STAT2 at tyrosine 690 (p-STAT2 Tyr690) demonstrated robust concentration- and time-dependent induction (Figure 3L). At Day 3, significant increases began at 600 µg (+1.86-fold) and rose to +2.35-fold at 1000 µg. At Day 6, phosphorylation was significantly induced starting at 200 µg (+2.47-fold) and reached +5.97-fold at 1000 µg. Statistical analysis confirmed significant effects of concentration and time, with a significant interaction term indicating enhanced phosphorylation with prolonged exposure.

As a result, PSP treatment was associated with reciprocal modulation of total STAT1 and STAT2 protein levels and their activated tyrosine-phosphorylated forms.

#### 2.2.5. Induction of IFN-γ Expression

PSP treatment resulted in a concentration- and time-dependent increase in cell-associated IFN-γ protein levels as assessed by immunoblotting of whole-cell lysates (Figure 3M). At Day 3, significant induction was detected beginning at 600 µg (+1.89-fold of the control) and remained elevated through 1000 µg (+1.91-fold). At Day 6, increased IFN-γ expression was observed at lower concentrations, beginning at 200 µg (+2.48-fold of the control) and progressively rising to +4.79-fold at 1000 µg. Two-way ANOVA demonstrated significant main effects of concentration and treatment duration, with enhanced magnitude of induction at Day 6.

IFN-γ was evaluated as an intracellular, cell-associated protein in whole-cell lysates. For the purpose of this study, secreted IFN-γ cytokine levels were not assessed.

### 2.3. PSP Induces Transcriptional Activation of Antiviral Signaling Genes in Jurkat T Cells

To determine whether the protein-level changes observed by immunoblotting were accompanied by transcriptional modulation, RT-qPCR analysis was performed at representative PSP concentrations (400, 600, and 1000 µg) at Days 3 and 6.

#### 2.3.1. TLR4 mRNA Expression

**Figure 4:**
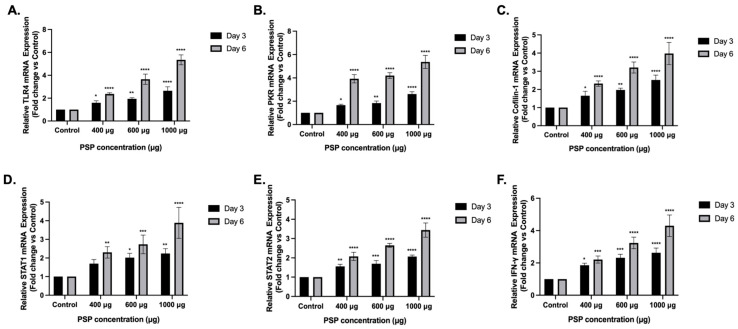
Transcriptional modulation of signaling and IFN-related genes in polysaccharide peptide (PSP)-treated Jurkat T cells. Relative messenger RNA (mRNA) expression levels of (**A**) Toll-like receptor 4 (TLR4), (**B**) Protein Kinase R (PKR), (**C**) Cofilin-1, (**D**) Signal transducer and activator of transcription (STAT) 1, (**E**) STAT2, and (**F**) Interferon gamma (IFN-γ) were determined by reverse transcription quantitative polymerase chain reaction (RT-qPCR) following PSP treatment at 400, 600, and 1000 µg/mL for 3 or 6 days. Transcript levels were normalized to 18S ribosomal RNA (rRNA) and expressed relative to untreated controls. Data are presented as mean ± standard deviation (SD) (n = 3 independent biological replicates). Statistical significance was determined using two-way analysis of variance (ANOVA) followed by Dunnett’s multiple comparisons test (vs. corresponding control). * *p* ≤ 0.05, ** *p* ≤ 0.01, *** *p* ≤ 0.001, **** *p* ≤ 0.0001.

PSP treatment significantly increased TLR4 mRNA expression in a concentration- and time-dependent manner (Figure 4A). At Day 3, significant induction was observed at 400 µg (+1.59-fold of the control), 600 µg (+1.94-fold), and 1000 µg (+2.64-fold). At Day 6, TLR4 transcripts were more strongly elevated, reaching +2.36-fold at 400 µg, +3.65-fold at 600 µg, and +5.35-fold at 1000 µg. Two-way ANOVA revealed significant main effects of concentration and treatment duration, with enhanced induction at prolonged exposure.

#### 2.3.2. PKR mRNA Expression

PKR mRNA levels were similarly upregulated following PSP treatment (Figure 4B). At Day 3, significant increases were detected at 400 µg (+1.66-fold), 600 µg (+1.84-fold), and 1000 µg (+2.62-fold). At Day 6, PKR transcripts exhibited stronger induction, reaching +3.92-fold at 400 µg, +4.20-fold at 600 µg, and +5.37-fold at 1000 µg. Statistical analysis confirmed significant concentration and time effects.

#### 2.3.3. Cofilin-1 mRNA Expression

Cofilin-1 transcripts were significantly elevated across the tested concentrations (Figure 4C). At Day 3, expression increased to +1.65-fold (400 µg), +1.97-fold (600 µg), and +2.52-fold (1000 µg). At Day 6, induction was amplified, reaching +2.32-fold, +3.20-fold, and +3.97-fold at 400, 600, and 1000 µg, respectively.

#### 2.3.4. STAT1 and STAT2 mRNA Expression

STAT1 mRNA expression demonstrated moderate but significant upregulation (Figure 4D). At Day 3, transcript levels increased to +1.69-, +2.01-, and +2.23-fold at 400, 600, and 1000 µg, respectively, with significance observed at 600 µg. At Day 6, STAT1 induction was more pronounced, reaching +2.30-fold, +2.73-fold, and +3.88-fold at the corresponding concentrations.

Similarly, STAT2 transcripts were significantly increased in response to PSP (Figure 4E). At Day 3, expression levels reached +1.56-fold (400 µg), +1.69-fold (600 µg), and +2.06-fold (1000 µg). At Day 6, STAT2 induction was further enhanced, increasing to +2.07-fold, +2.64-fold, and +3.43-fold, respectively.

#### 2.3.5. IFN-γ mRNA Expression

To evaluate whether PSP modulates antiviral cytokine signaling at the transcriptional and intracellular protein levels, IFN-y mRNA expression was assessed following PSP treatment.

IFN-γ mRNA expression was significantly elevated following PSP treatment (Figure 4F). At Day 3, transcripts increased to +1.85-fold (400 µg), +2.32-fold (600 µg), and +2.63-fold (1000 µg). At Day 6, IFN-γ induction was substantially higher, reaching +2.32-, +3.23-, and +4.30-fold at 400, 600, and 1000 µg, respectively. Two-way ANOVA confirmed significant effects of concentration and treatment duration.

Taken together, these results demonstrate that PSP induces coordinated transcriptional activation of multiple antiviral signaling genes in Jurkat T cells, consistent with the protein-level changes observed by immunoblotting.

## 3. Discussion

Building upon our previous findings in THP-1 monocytic cells, where PSP restricted HIV-1 entry through a PKR-dependent mechanism, the present study sought to determine whether PSP induces similar antiviral-associated signaling programs in Jurkat CD4+ T cells. Because CD4+ T cells represent primary targets of HIV-1 infection, understanding whether PSP modulates key IFN, PKR, NF-κB, and cytoskeletal pathways in this cellular context is essential before directly evaluating viral permissiveness. Accordingly, this study was designed to establish whether PSP elicits robust, concentration- and time-dependent molecular regulation in Jurkat T cells without inducing cytotoxicity, thereby providing a mechanistic foundation for subsequent studies.

Consistent with this objective, PSP treatment resulted in progressive upregulation of TLR4, PKR, phospho-PKR (Thr446), Cofilin-1, phospho-Cofilin-1 (Ser3), STAT1, phospho-STAT1 (Tyr701), STAT2, phospho-STAT2 (Tyr690), and IFN-γ at both the protein and transcript levels. Importantly, these signaling changes were amplified at prolonged exposure (Day 6), suggesting that sustained PSP exposure enhances immunoregulatory programming in Jurkat T cells. These findings are consistent with our previous observations in THP-1 monocytic cells [9] and support the notion that PSP induces conserved antiviral-associated signaling programs across two immune cell types. Although HIV-1 infection was not directly assessed in the present work, the observed parallel signaling architecture is consistent with modulation of pathways associated with antiviral responses.

PSP has previously been reported to engage TLR4-associated signaling pathways in immune cells, leading to modulation of downstream NF-κB- and IFN-related responses [7,8,9,15,16,17]. A key study has demonstrated that PSP and related *Coriolus versicolor*-derived polysaccharides can influence TLR4-dependent transcriptional programs in monocytic and macrophage models [18], supporting a role for TLR4 as an upstream immunomodulatory node.

Importantly, TLR4 protein levels were assessed in whole-cell lysates, reflecting total cellular protein abundance rather than surface receptor expression. The present study does not evaluate membrane localization or receptor trafficking. Instead, the data indicate increased intracellular TLR4 protein levels following PSP exposure. Our findings are consistent with these observations at the level of total cellular protein expression. The observed induction of TLR4 in Jurkat T cells aligns with prior reports of PSP-mediated innate immune pathway activation and provides mechanistic continuity with previously described TLR4-NF-κB-IFN signaling cascades. However, we do not claim direct receptor engagement or surface activation in this model.

TLR4 is a well-characterized pattern recognition receptor that activates downstream transcriptional programs through MyD88-dependent and TRIF-dependent pathways, culminating in NF-κB and IFN regulatory factor activation [19,20]. Engagement of TLR4 is known to lead to phosphorylation of the NF-κB p65 subunit at serine 536, enhancing transcriptional activity [20]. Given previous reports indicating that PSP modulates TLR4-associated responses in immune cells [8,9,19], the coordinated increase in intracellular TLR4 levels and NF-κB phosphorylation observed here supports activation of a TLR4-linked transcriptional axis in Jurkat T cells.

NF-κB is a central regulator of immune-responsive genes, including cytokines, IFN, and interferon-stimulated genes (ISGs) [21]. Activation of NF-κB can contribute to transcription of IFN-γ and other antiviral mediators in T cells [22]. In our study, NF-κB signaling exhibited a bidirectional regulatory pattern. Total NF-κB protein levels decreased, whereas phospho-NF-κB (Ser536) was significantly increased. Phosphorylation at Ser536 enhances transcriptional activity of the p65 subunit [23,24]. This pattern may reflect selective activation of NF-κB signaling, where a greater fraction of NF-κB is maintained in its transcriptionally active, phosphorylated state despite reduced total protein abundance. In addition, phosphorylation of NF-κB is associated with nuclear translocation and transcriptional engagement, processes that may reduce the detectable cytoplasmic pool of total NF-κB protein [23,24]. Furthermore, activation-dependent turnover and tightly regulated feedback mechanisms may contribute to decreased steady-state levels of total NF-κB despite enhanced signaling activity. Such dynamics are consistent with regulated inflammatory signaling pathways, where activation state rather than total protein abundance determines transcriptional output. Previous studies have implicated TLR4-mediated NF-κB activation in PSP signaling contexts [8,9,18]. However, as with TLR4, the present work measures intracellular protein abundance and phosphorylation state rather than receptor engagement dynamics.

STAT1 (Tyr701) and STAT2 (Tyr690) phosphorylation are hallmark activation events within IFN signaling cascades [25]. Upon cytokine engagement, STAT1 and STAT2 undergo tyrosine phosphorylation, form transcriptional complexes, and promote expression of ISG to promote antiviral and anti-HIV-1 responses [25,26]. Phosphorylation at these tyrosine residues is required for STAT activation and transcriptional complex formation [25]. In this study, the STAT pathway was also markedly modulated. Total STAT1 and STAT2 protein levels were reduced in a dose- and time-dependent manner, while phosphorylation of STAT1 (Tyr701) and STAT2 (Tyr690) was significantly increased. The observed divergence between total STAT abundance and phosphorylated forms suggests dynamic regulation favoring activation over accumulation. Enhanced phosphorylation at Day 6 indicates sustained engagement of the IFN pathway under prolonged PSP exposure. This apparent divergence may reflect preferential activation of signaling pools, whereby a greater proportion of STAT1 and STAT2 is maintained in an active, phosphorylated state under PSP exposure. In addition, activation-dependent nuclear translocation and increased protein turnover may contribute to reduced steady-state levels of total protein despite enhanced signaling activity. Such dynamics are consistent with tightly regulated cytokine signaling pathways, where activation state rather than total protein abundance dictates downstream transcriptional responses.

Because IFN-γ signaling can further amplify IFN-responsive pathways and promote expression of antiviral genes such as PKR, IFN-γ was included in this study as an indicator of cytokine-driven antiviral immune activation. The observed increase in intracellular IFN-γ protein and mRNA following PSP exposure is consistent with enhanced immune transcriptional activity downstream of NF-κB activation. Consistent with STAT activation, intracellular IFN-γ protein levels were significantly elevated in PSP-treated Jurkat cells. IFN-γ was measured as a cell-associated protein in whole-cell lysates; secreted cytokine levels were not assessed in this study. Thus, for the main purpose of this study, the present findings reflect intracellular accumulation rather than extracellular cytokine release. However, it is known that IFN-γ is a central effector cytokine in T-cell-mediated antiviral immunity and contributes to the induction of ISGs, including PKR [27]. While intracellular IFN-γ accumulation supports activation of cytokine-associated transcriptional programs, it does not directly reflect cytokine secretion or extracellular signaling activity. Therefore, the present findings should be interpreted as indicative of intracellular immune activation rather than definitive evidence of a functional cytokine-mediated antiviral state. Future studies assessing secreted IFN-γ levels and downstream receptor engagement will be necessary to fully establish cytokine-driven antiviral activity. The coordinated induction of IFN-γ, STAT phosphorylation, and PKR expression observed here suggests the establishment of an antiviral-leaning transcriptional environment in Jurkat cells.

Downstream of TLR4, PSP treatment was associated with significant upregulation of PKR and phospho-PKR (Thr446). PKR is a well-characterized IFN-inducible kinase implicated in antiviral defense, HIV-1 restriction, and inhibition of HIV-1 replication [28]. Phosphorylation at Thr446 is associated with PKR activation and downstream substrate regulation [29]. The robust, time and dose-dependent induction of PKR observed in Jurkat cells mirrors the signaling profile previously documented in our study using PSP-treated monocytic cells [30], reinforcing the hypothesis that PKR is a central node in PSP-mediated immunomodulation. The coordinated upregulation of TLR4, NF-κB phosphorylation, STAT1/STAT2 phosphorylation, PKR expression, and p-PKR (Thr446) observed in the present study supports the existence of an integrated IFN-associated signaling cascade induced by PSP. This is particularly relevant given our prior demonstration that PKR activation contributes to Cofilin-1 phosphorylation and HIV-1 entry restriction in monocytic cells [9].

Cofilin-1 and phospho-Cofilin-1 (Ser3) were similarly upregulated. Phosphorylation at Ser3 inhibits Cofilin-1 actin-severing activity, thereby altering actin cytoskeletal dynamics [31]. Cytoskeletal remodeling is critically involved in T-cell activation, immune synapse formation, and viral entry processes [32]. In the context of our prior HIV-1 study, significant upregulation of PSP-mediated PKR activation was associated with Cofilin-1 phosphorylation, resulting in sustained actin polymerization dynamics that reduced viral entry by approximately 73% [9]. Although viral assays were not performed here, the replication of this PKR-Cofilin axis in Jurkat cells suggests that PSP induces cytoskeletal regulatory programs that are known to regulate cytoskeletal dynamics involved in viral entry processes [9].

**Figure 5:**
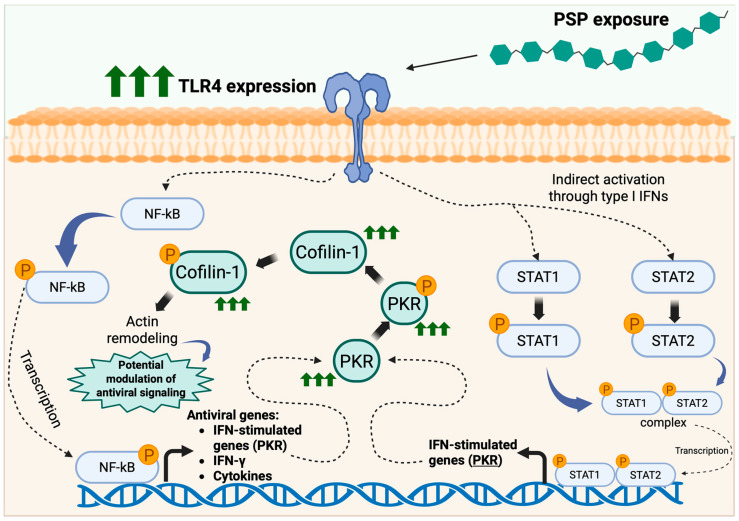
Proposed model of polysaccharide peptide (PSP)-induced antiviral signaling and cytoskeletal regulation in Jurkat T cells. Exposure of Jurkat T cells to PSP derived from *Coriolus versicolor* was associated with increased toll-like receptor 4 (TLR4) expression and activation of downstream innate immune signaling pathways. Elevated TLR4 expression is proposed to promote activation of the nuclear factor kappa B (NF-κB) pathway, leading to NF-κB phosphorylation and nuclear translocation, and the transcription of antiviral and inflammatory genes, including interferon gamma (IFN-γ) and interferon-stimulated genes (ISG). In parallel, TLR4 signaling may indirectly promote the activation of signal transducer and activator of transcription (STAT) 1 and STAT2 via type I IFN–associated pathways. Phosphorylated STAT1 and STAT2 form transcriptionally active complexes that translocate to the nucleus and induce expression of ISG, including Protein Kinase R (PKR). Increased PKR expression and phosphorylation may contribute to the regulation of cytoskeletal dynamics through the modulation of Cofilin-1 activity, promoting actin remodeling. These signaling events are consistent with pathways previously implicated in antiviral responses, including those relevant to HIV-1 biology, as reported in our prior study [9]. Arrows indicate proposed signaling relationships based on the protein and transcriptional changes observed in this study. Green arrows indicate increased expression or activation of signaling components. Created in BioRender. Alvarez-Rivera, E. (2026) https://BioRender.com/vk9xld2 (accessed on 10 April 2026).

Collectively, the markers selected in this study represent interconnected nodes within a coherent antiviral signaling network. TLR4-associated signaling can activate NF-κB and IFN pathways; NF-κB and IFN signaling promote STAT1/STAT2 activation; activated STAT complexes drive transcription of ISG, including PKR; and PKR activation can influence cytoskeletal regulators such as Cofilin-1 [9,33,34]. Thus, the molecular markers analyzed in this work were selected to evaluate sequential components of a TLR4-NF-κB-STAT-PKR-Cofilin axis that is mechanistically relevant to antiviral host defense. Based on the coordinated molecular changes observed in this study, a proposed mechanistic model summarizing PSP-associated antiviral signaling in Jurkat T cells is presented in Figure 5.

Notably, PSP treatment did not significantly reduce Jurkat T-cell viability at any of the tested concentrations. MTT, trypan blue exclusion, and viable cell density analyses demonstrated preservation of metabolic activity, membrane integrity, and proliferative capacity, respectively. These findings are critical, as they indicate that the observed signaling changes are not secondary to cytotoxic stress but instead reflect active immunomodulatory regulation. Interestingly, a modest increase in viable cell density was observed at intermediate PSP concentrations (400–600 μg/mL), particularly at prolonged exposure. While this study was not designed to directly assess proliferation, this observation may reflect enhanced cellular metabolic activity or survival signaling under PSP-induced immunomodulatory conditions. Such effects have been reported for certain immunomodulatory polysaccharides, which can promote immune cell activation and functional responsiveness without inducing uncontrolled proliferation [35,36].

In this context, increased viable cell density may contribute to a more robust antiviral signaling environment by supporting sustained activation of key pathways such as NF-κB, STAT, and PKR. However, further studies specifically designed to assess proliferation and cell cycle dynamics will be required to distinguish between increased survival, metabolic activity, and proliferative responses.

Taken together, the present study demonstrates that PSP induces coordinated activation of TLR4-associated, PKR-mediated, STAT-dependent, and IFN-related pathways in Jurkat T cells in a dose- and time-dependent manner. These signaling profiles closely parallel those previously observed in monocytic models, supporting the concept that PSP modulates conserved antiviral regulatory networks across immune cell lineages.

In addition to the signaling pathways characterized in this study, consideration of potential off-target interactions is important for the broader pharmacological evaluation of PSP. In particular, interactions with drug-metabolizing enzymes, such as cytochrome P450 (CYP) isoforms, are typically assessed in hepatic and in vivo systems rather than in lymphoid cell models. Given that the present study was conducted in Jurkat T cells, which are not specialized for xenobiotic metabolism, evaluation of CYP-mediated interactions falls outside the scope of this experimental system.

Furthermore, PSP is a high-molecular-weight protein-bound polysaccharide, approximately 100 kDa in crude extract form [37], and is thought to exert its biological effects primarily through interactions with cell surface receptors, including TLR4, rather than through intracellular metabolic processing. Consistent with this, previous in vivo studies have reported that PSP does not significantly alter CYP-mediated drug metabolism, including no measurable impact on tamoxifen metabolism or associated biochemical parameters [38]. These observations suggest that PSP may have limited interaction with CYP-dependent metabolic pathways; however, comprehensive pharmacokinetic and ADME studies will be required to fully characterize potential off-target effects in physiologically relevant systems.

While HIV-1 is referenced due to the well-established involvement of the analyzed signaling pathways in viral entry and replication, the present study was not designed to directly assess viral infectivity or replication. Instead, this work focuses on defining the intracellular signaling landscape induced by PSP in CD4+ T cells as a mechanistic foundation for future functional studies. Direct HIV-1 infection assays were beyond the scope of this study; the mechanistic overlap with our previously published PSP-mediated restriction of HIV-1 entry in THP-1 cells provides a rationale for our future investigations in adaptive CD4+ T-cell models. The present work establishes that PSP can modulate key antiviral signaling nodes in Jurkat cells without inducing cytotoxicity, thereby laying the foundation for subsequent studies evaluating HIV-1 permissiveness and viral replication in PSP-conditioned T cells.

These findings contribute mechanistic insight into how natural immunomodulators may prime antiviral signaling pathways in CD4+ T-cell models. By delineating concentration- and time-dependent activation of PKR, STAT, NF-κB, and IFN-γ pathways in Jurkat cells, this study provides a translational bridge between innate immune modulation and adaptive antiviral preparedness.

## 4. Materials and Methods

### 4.1. Cell Culture

Neo Jurkat cells (ATCC^®^ CRL-2898™, a T lymphocyte cell line derived from human acute T-cell leukemia) were obtained from the American Type Culture Collection (ATCC, Manassas, VA, USA). Cells were maintained in ATCC-formulated RPMI-1640 medium supplemented with 10% heat-inactivated fetal bovine serum (FBS), 100 U/mL penicillin, and 100 µg/mL streptomycin. A concentration of 200 µg/mL G418 was included in the culture medium to maintain selection pressure.

Cells were grown in suspension culture in a T-75 cm^2^ culture flask, at 37 °C using a humidified incubator with 5% carbon dioxide (CO_2_) and passaged every 3 days to maintain a density of 2 × 10^5^–2 × 10^6^ cells/mL.

### 4.2. PSP Extraction and Treatment

PSP was extracted from commercially available PSP supplement tablets (Mushroom Science, Eugene, OR, USA), standardized to contain a 28% polysaccharide-to-peptide ratio and 60.23 mg/g β-1,3/1,6-glucan. Extraction was performed using a previously established protocol from our prior published work [9], ensuring methodological consistency with the experimental systems used previously. This extraction approach has also been successfully applied in other human monocytic THP-1 cell models by independent studies [8].

PSP tablets were dissolved in sterile, endotoxin-free water (Sigma-Aldrich, St. Louis, MO, USA) preheated to 90–100 °C under aseptic conditions, then centrifuged at 2060× *g* for 5 min to remove insoluble material. The extraction cycle was repeated until the supernatant was clear of visible residues. The aqueous extract was mixed with 80% ethanol to precipitate the polysaccharide-rich fraction, and the light-brown PSP-containing fraction was collected. The precipitate was washed with absolute ethanol, centrifuged, and dried using a refrigerated vapor trap (Thermo Fisher Scientific, Waltham, MA, USA) at −105 °C. The dried extract was reconstituted in sterile, endotoxin-free water and stored at −20 °C until use.

For experimental treatments, Jurkat T cells were exposed to PSP at concentrations ranging from 50 to 1000 μg/mL to assess dose- and time-dependent responses. These concentrations were selected based on prior studies demonstrating immunomodulatory activity at intermediate doses (stated at 200 μg/mL) [8,9], and were expanded to include lower and higher concentrations to establish a dose–response profile. The upper concentration (1000 μg/mL) was included to assess maximal cellular exposure and confirm that PSP-induced signaling changes occur in the absence of cytotoxic effects. Given the complex composition and limited pharmacokinetic characterization of PSP, precise pharmacologically relevant concentrations in vivo remain incompletely defined. Therefore, the concentration range used in this study was designed to capture a broad spectrum of biological responses under controlled in vitro conditions rather than to directly model physiological exposure levels. PSP treatment was initiated on Day 0, and cells were harvested and analyzed at two experimental time points (Day 3 and Day 6), which were selected to capture both early and sustained signaling responses as part of the time-dependent design of the study. The inclusion of a 6-day time point was further guided by prior work evaluating PSP-mediated effects in monocytic cells, enabling consistency in temporal analysis across studies [9]. Medium was replenished on Day 3 in both control and PSP-treated cultures to maintain consistent nutrient availability, reduce waste accumulation and overgrowth-related stress, and preserve stable culture conditions during the 6-day exposure period. Cells were harvested and analyzed at the indicated timepoints depending on the downstream assay. Cells were maintained at 37 °C in a humidified incubator with 5% CO_2_ throughout the experimental period.

### 4.3. MTT Cell Viability Assay

Cell viability following PSP treatment was assessed using the MTT assay (Sigma-Aldrich, St. Louis, MO, USA), according to the manufacturer’s protocol.

Jurkat T cells were seeded at a density of 1 × 10^4^ cells per well in 96-well plates in biological and technical triplicates and allowed to equilibrate for 24 h at 37 °C in a humidified incubator with 5% CO_2_. Cells were then treated with PSP at concentrations ranging from 50 to 1000 µg/mL at day 0 and replenished at day 3 to mirror the experimental design used in downstream signaling analyses. Cell viability was assessed at two experimental time points (Day 3 and Day 6).

On day 3 or 6, the 5 mg/mL MTT solution prepared in 1× phosphate-buffered saline (PBS) was added to each well and incubated for 4 h at 37 °C with 5% CO_2_ to allow the formation of formazan crystals. After incubation, the supernatant was carefully removed, and the crystals were solubilized in 100% dimethyl sulfoxide (DMSO). Absorbance was measured at 570 nm using a Molecular Devices VersaMax microplate reader (GMI Trusted Laboratory Solutions, Ramsey, MN, USA).

Cell viability was calculated as the percentage of treated cells relative to positive and negative control groups. All experiments were performed in at least three independent biological replicates.

### 4.4. Trypan Blue Exclusion Assay

To validate cell viability independently of mitochondrial metabolic activity, trypan blue exclusion assays were performed following PSP treatment.

Jurkat T cells were seeded at a density of 5 × 10^5^ cells per well in 6-well plates and treated with PSP at concentrations ranging from 50 to 1000 µg/mL beginning on day 0. For extended exposure experiments, PSP-containing medium was replenished on Day 3 to maintain treatment consistency with downstream signaling analyses.

Cell viability was assessed at two experimental time points (Day 3 and Day 6). At the indicated time points, cells were collected, gently resuspended, and mixed 1:10 with 0.4% trypan blue solution (Sigma-Aldrich, St. Louis, MO, USA). Viable (unstained) and non-viable (blue-stained) cells were manually counted using a hemocytometer under light microscopy.

Cell counts were performed immediately after staining to minimize dye uptake artifacts. All measurements were performed in biological triplicates, and cell viability was calculated as the percentage of viable cells relative to the total number of cells counted.

### 4.5. Quantification of Viable Cell Density

In addition to viability assessment by MTT and trypan blue exclusion, total viable cell density was quantified to determine whether PSP impacts Jurkat T-cell density.

Jurkat T cells were plated at the same density (5 × 10^5^ cells per well in 6-well plates) and treated with PSP at concentrations ranging from 50–1000 µg/mL starting on Day 0 to maintain consistency with the trypan blue exclusion assays. For extended exposure experiments, the PSP-containing medium was refreshed on Day 3 in alignment with downstream signaling analyses.

Viable cell density was assessed at two experimental time points (Day 3 and Day 6). At the indicated time points, cells were harvested and mixed 1:10 with 0.4% trypan blue solution (Sigma-Aldrich, St. Louis, MO, USA). Viable (unstained) cells were counted using a hemocytometer, and total viable cells per milliliter were calculated.

Viable cell density for each treatment group was normalized to the untreated control, which was set to 100%. Results are presented as a percentage of the control to assess relative changes in viable cell expansion following PSP exposure. All quantification experiments were performed in biological triplicate.

### 4.6. Western Blot

Protein lysates were collected from PSP-treated Jurkat T cells at Days 3 and 6 following treatments. Total protein was extracted from Jurkat T cells using radioimmunoprecipitation assay (RIPA) lysis buffer (Thermo Fisher Scientific, Waltham, MA, USA) supplemented with a protease and phosphatase inhibitor cocktail (Thermo Fisher Scientific, Waltham, MA, USA) and lysates clarified by centrifugation. Protein concentration was determined using a Pierce Bicinchoninic acid (BCA) protein assay kit (Thermo Fisher Scientific, Waltham, MA, USA) and spectrophotometrically in a Molecular Devices VersaMax Absorbance Microplate Reader (GMI Trusted Laboratory Solutions, Ramsey, MN, USA). Equal amounts of protein (30 μg per sample) were resolved by sodium dodecyl sulfate polyacrylamide gel electrophoresis (SDS-PAGE) and transferred onto polyvinylidene difluoride (PVDF) membranes.

Membranes were blocked in 5% bovine serum albumin (BSA; Fisher Scientific, Hampton, NH, USA) prepared in 1× tris-buffered saline with tween 20 (TBST), followed by overnight incubation with primary antibodies at 4 °C with gentle agitation. Primary antibodies were used at 1:1000 dilution unless otherwise specified. The following targets were analyzed: cofilin-1, phospho-cofilin-1 (Ser3), PKR, phospho-PKR (Thr446), TLR4, NF-κB p65, phospho-NF-κB p65 (Ser536), STAT1, phospho-STAT1 (Tyr701), STAT2, phospho-STAT2 (Tyr690), and IFN-γ. All primary antibodies were obtained from Cell Signaling Technology (Danvers, MA, USA) except phospho-PKR (Thr446), which was obtained from Abcam (Cambridge, UK). β-Actin (Cell Signaling Technology, Danvers, MA, USA) was used as the loading control.

After primary incubation, membranes were washed in 1× TBST and incubated with horse radish peroxidase (HRP)-conjugated secondary antibodies (Cell Signaling Technology, Denvers, MA, USA) at 1:10,000 dilution. Protein bands were detected by enhanced chemiluminescence using the SuperSignal West Femto Maximum Sensitivity Substrate Kit (Thermo Fisher Scientific, Waltham, MA, USA) and visualized using a Molecular Imager^®^ ChemiDoc™ XRS+ Imaging System (Bio-Rad Laboratories, Hercules, CA, USA). Densitometric analysis was performed using ImageJ software version 1.54g (National Institutes of Health, Bethesda, MD, USA). All experiments were performed in three independent biological replicates.

### 4.7. Reverse Transcription Quantitative Polymerase Chain Reaction (RT-qPCR)

RT-qPCR analysis was performed at representative PSP concentrations (400, 600, and 1000 µg/mL) in Jurkat T cells at Day 3 and Day 6. These concentrations were selected based on robust protein-level modulation observed in immunoblot analyses. Total RNA was isolated from Jurkat T-cell pellets using the RNeasy Plus Mini Kit (Qiagen, Hilden, Germany) according to the manufacturer’s instructions. RNA concentration and purity were assessed spectrophotometrically using an Accuris NS1000 SmartDrop L Nano Spectrophotometer (Accuris Instruments, Edison, NJ, USA). Complementary DNA (cDNA) was synthesized from 1 µg of total RNA using the iScript cDNA Synthesis Kit (Bio-Rad, Hercules, CA, USA).

Quantitative PCR was performed using SYBR Green chemistry (Bio-Rad, Hercules, CA, USA) with gene-specific primers at a final concentration of 500 nM. Amplification reactions were run on a Bio-Rad CFX96 Touch Real-Time PCR Detection System (Bio-Rad, Hercules, CA, USA) under the following cycling conditions: 95 °C for 15 s, at the gene-specific annealing temperature for 1 min, and 72 °C for 1 min, for a total of 40 cycles.

Target genes analyzed included cofilin-1, PKR, TLR4, STAT1, STAT2, and IFN-γ. The primer sequences used were as follows (based on published sequences and custom synthesized by Sigma-Aldrich, St. Louis, MO, USA): cofilin-1 forward, 5′-CCTCCATCCCTTGACGGTTC-3′ and reverse, 5′-GTGGGGAATGGGGATGTTGT-3′; PKR forward, 5′-GAAGTGGACCTCTACGCTTTGG-3′ and reverse, 5′-TGATGCCATCCCGTAGGTCTGT-3′; TLR4 forward, 5′-CCCTGAGGCATTTAGGCAGCTA-3′ and reverse, 5′-AGGTAGAGAGGTGGCTTAGGCT-3′; STAT1 forward, 5′-ATGGCAGTCTGGCGGCTGAATT-3′ and reverse, 5′-CCAAACCAGGCTGGCACAATTG-3′; STAT2 forward, 5′-CAGGTCACAGAGTTGCTACAGC-3′ and reverse, 5′-CGGTGAACTTGCTGCCAGTCTT-3′; IFN-γ forward, 5′-GAGTGTGGAGACCATCAAGGAAG-3′ and reverse, 5′-TGCTTTGCGTTGGACATTCAAGTC-3′.

Relative gene expression was determined using the comparative cycle threshold (Ct) method (2^−ΔΔCt^). Expression levels were normalized to 18S rRNA (forward, 5′-GGCCCTGTAATTGGAATGAGTC-3′; reverse, 5′-CCAAGATCCAACTACGAGCTT-3′), which served as the endogenous control. All experiments were performed in at least three independent biological replicates.

### 4.8. Statistical Analysis

All data are presented as the mean ± standard deviation (SD) from biological triplicates. Statistical analyses were performed using GraphPad Prism version 10.2.0 (GraphPad Software, San Diego, CA, USA). Differences among groups were evaluated using two-way analysis of variance (ANOVA), followed by Dunnett’s multiple comparisons test to compare each group’s mean with the corresponding control mean within each column. A *p*-value ≤ 0.05 was considered statistically significant.

## 5. Study Limitations

While the present study provides a comprehensive analysis of PSP-induced signaling modulation in Jurkat T cells and was designed as a mechanistic and descriptive investigation, several limitations should be acknowledged. First, this work focuses on molecular and transcriptional changes and does not include direct functional validation of antiviral activity, such as viral infectivity or replication assays. As such, the observed signaling alterations should be interpreted as indicative of a primed antiviral signaling environment rather than direct evidence of viral restriction.

Second, the study was conducted using a single transformed CD4+ T-cell line (Jurkat), which may not fully recapitulate the complexity of primary human T cells. Validation in additional cellular models, including primary CD4+ T lymphocytes, will be necessary to confirm the broader physiological relevance of these findings.

Finally, while the data support coordinated modulation of TLR4-associated, NF-κB, STAT, and PKR signaling pathways, the precise upstream mechanisms and potential off-target intracellular interactions of PSP remain to be fully elucidated. Future studies incorporating targeted pathway inhibition and broader molecular profiling will be required to define these mechanisms in greater detail. In addition, the present study does not account for the pharmacokinetic properties of PSP, including its in vivo half-life, stability, and bioavailability. Therefore, the sustained exposure conditions used in this in vitro model may not directly reflect physiological PSP dynamics in vivo, and further studies will be required to define its pharmacokinetic and translational profile. Importantly, the present study does not establish a causal relationship between PSP-induced signaling changes and antiviral activity, and no conclusions regarding viral inhibition can be drawn from these data.

## 6. Conclusions

In conclusion, PSP treatment induces coordinated, concentration- and time-dependent activation of TLR4-associated, NF-κB-regulated, STAT-dependent, and PKR-mediated signaling pathways in Jurkat T cells without compromising cellular viability. The observed induction of phospho-PKR (Thr446) and phospho-Cofilin-1 (Ser3), alongside activation of STAT1/STAT2 and intracellular IFN-γ upregulation, supports the establishment of a transcriptional profile consistent with activation of IFN-associated signaling pathways in adaptive CD4+ T-cell models. These findings extend our prior observations in monocytic cells and demonstrate that PSP modulates conserved immune signaling nodes across immune lineages. Importantly, the absence of cytotoxicity confirms that these effects reflect active immunoregulatory programming rather than stress-induced responses. This work provides mechanistic evidence that PSP primes antiviral signaling architecture in Jurkat T cells and establishes a foundation for functional viral permissiveness studies. Further studies will be required to define the full spectrum of PSP-mediated intracellular interactions and to determine how these signaling effects integrate with broader cellular regulatory networks.

## 7. Future Directions

Although direct HIV-1 infection assays were beyond the scope of this study, the conserved PKR–Cofilin signaling axis observed here, which has previously been linked in our studies to HIV-1 entry restriction in THP-1 monocytic cells, provides a strong mechanistic rationale for subsequent investigation. Our future research will evaluate whether PSP-conditioned Jurkat T cells exhibit reduced HIV-1 entry and replication, and whether pharmacologic or genetic inhibition of PKR and upstream signaling nodes attenuates PSP-mediated effects. In addition, to further strengthen the physiological relevance of these findings, future work will extend these analyses to additional CD4+ T-cell models, including primary human T lymphocytes. These approaches will allow us to determine whether the antiviral signaling framework defined here translates into functional viral restriction in more physiologically relevant adaptive immune systems. Furthermore, future studies will incorporate broader mechanistic profiling approaches to evaluate potential PSP interactions with additional intracellular regulatory pathways, including those not directly assessed in the present study.

## Data Availability

The data supporting the results are presented in the manuscript and the Supplementary Materials. Uncropped Western blot images are provided as Supplementary Figures. Additional raw data supporting the findings of this study are available from the corresponding author upon request.

## Supplementary Materials

The following supporting information can be downloaded at: https://www.mdpi.com/article/10.3390/ijms27083661/s1.

## Abbreviations

The following abbreviations are used in this manuscript:

ANOVA	Analysis of variance
ATCC	American type culture collection
BCA	Bicinchoninic acid
BSA	Bovine serum albumin
CD4	Cluster of differentiation 4
cDNA	Complementary DNA
CO_2_	Carbon dioxide
Ct	Cycle threshold
DMSO	Dimethyl sulfoxide
FBS	Fetal bovine serum
HIV-1	Human immunodeficiency virus type 1
HRP	Horseradish peroxidase
IFN	Interferon
IFN-γ	Interferon gamma
ISGs	Interferon-stimulated genes
mRNA	Messenger ribonucleic acid
MTT	Thiazolyl blue tetrazolium bromide
NF-κB	Nuclear factor kappa B
PBS	Phosphate-buffered saline
PKR	Protein kinase R
PSP	Polysaccharide peptide
PVDF	Polyvinylidene difluoride
RIPA	Radioimmunoprecipitation assay buffer
RNA	Ribonucleic acid
RT-qPCR	Reverse transcription quantitative polymerase chain reaction
rRNA	Ribosomal ribonucleic acid
SDS-PAGE	Sodium dodecyl sulfate polyacrylamide gel electrophoresis
SD	Standard deviation
STAT	Signal transducer and activator of transcription
STAT1	Signal transducer and activator of transcription 1
STAT2	Signal transducer and activator of transcription 2
TBST	Tris-buffered saline with tween 20
TLR4	Toll-like receptor 4
