# Supplementary material for: Time- and Dose-Dependent PSP-Induced Modulation of Antiviral Signaling Networks in CD4+ T Cells
Source: Int J Mol Sci. 2026 Apr 20;27(8):3661. doi: 10.3390/ijms27083661 (PMC13115976; doi:10.3390/ijms27083661)

Figure S1: This supplementary figure contains the original, uncropped western blot images corresponding to the representative blots shown in the main manuscript. Lane order corresponds to the experimental conditions indicated in the main figures. Images are grouped according to experimental time point (PSP Day 3 and PSP Day 6). In some images, brightness and contrast were uniformly adjusted using Bio-Rad Image Lab software solely to improve visualization of the PVDF membrane background. These adjusted images were not used for densitometric quantification; all quantitative analyses were performed using the original, unprocessed images. Adjustments were applied equally to the entire image and did not alter the relative intensities or distributions of the protein bands. Due to the strong chemiluminescent signal and short exposure times, the PVDF membrane background may appear faint in some images.

Instrument used for image acquisition: Molecular Imager® ChemiDoc™ XRS+ Imaging System (Bio-Rad Laboratories, Hercules, CA, USA) using Image Lab software.

Molecular weight marker used: Precision Plus Protein™ All Blue Prestained Protein Standards (Bio-Rad Laboratories).

Section 1: Western blot photos found in Figure 2 corresponding for PSP Day 3

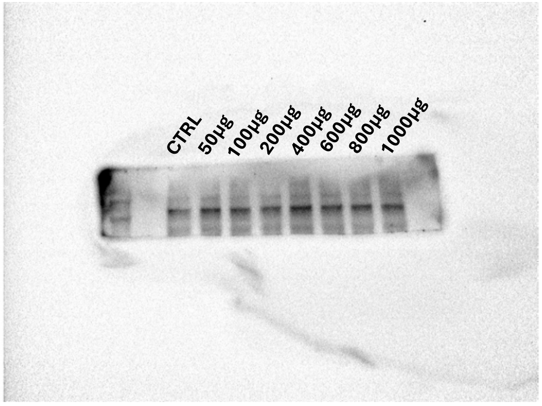

TLR4
120-140kDa

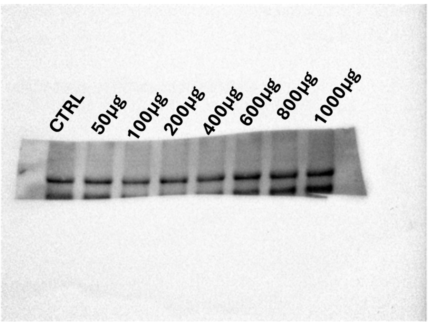

PKR
74kDa

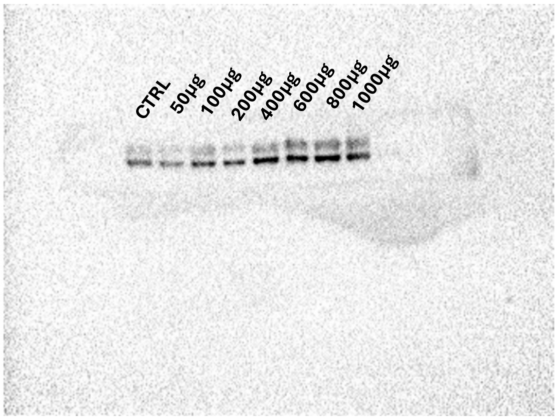

p-PKR (T446)
62-68kDa

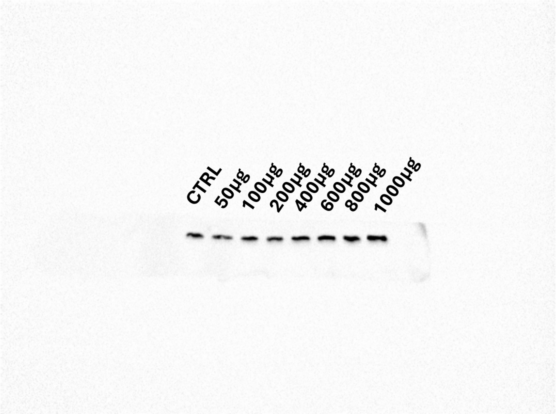

Cofilin-1
19kDa

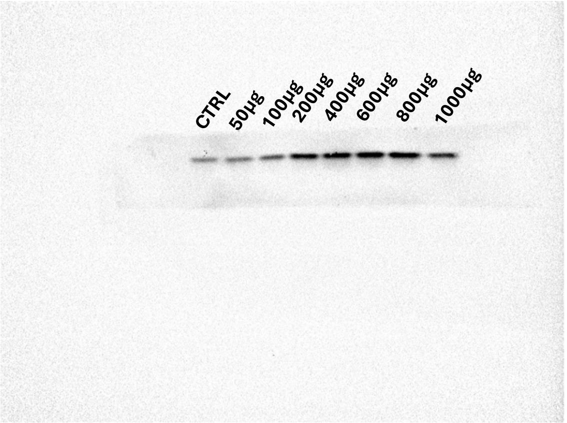

p-Cofilin-1 (Ser 3)
19kDa

Original representative image of NF-κB found within the manuscript.

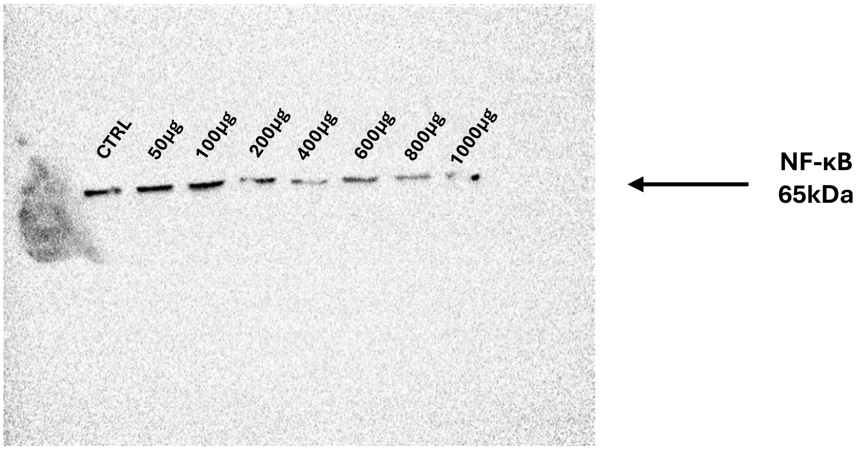

Same photo of NFκB with contrast adjusted using Bio-Rad Image Lab software:

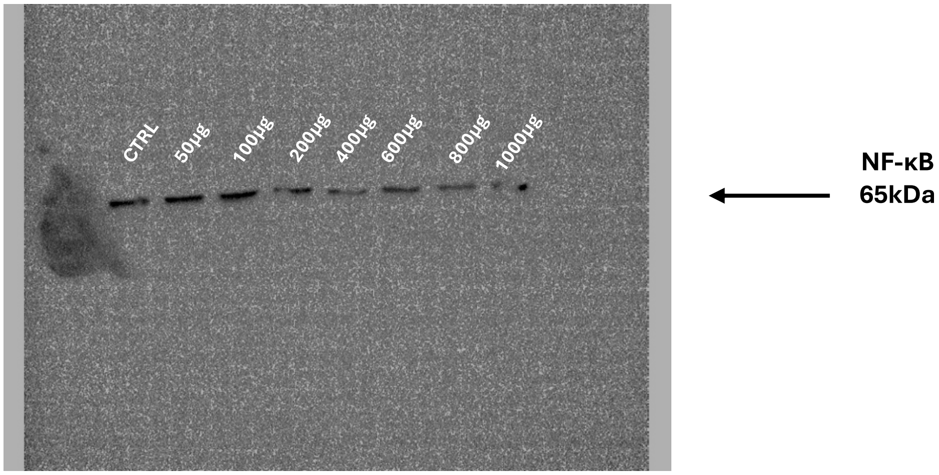

Original representative image of p-NF-κB (Ser 536, p65) found within the manuscript.

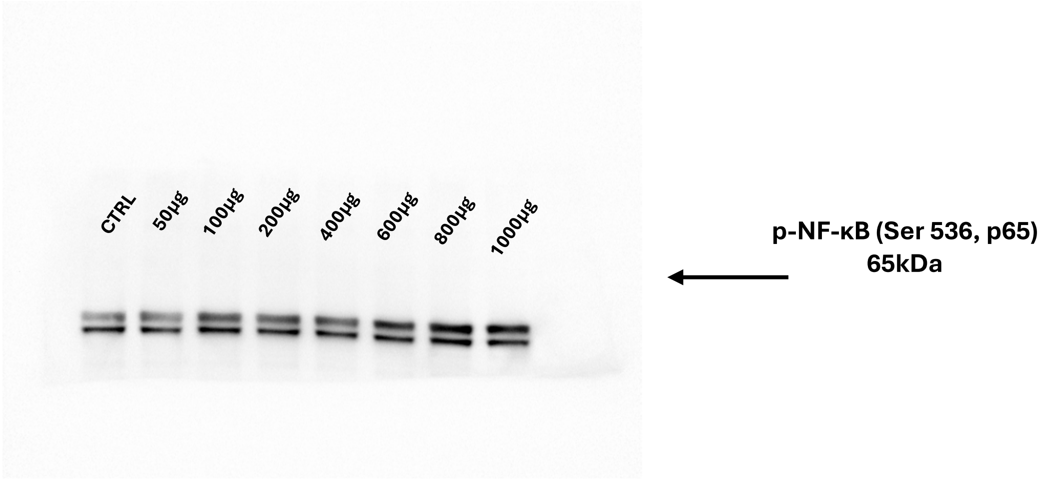

Same photo of p-NFκB (Ser 536, p65) with contrast adjusted using Bio-Rad Image Lab software:

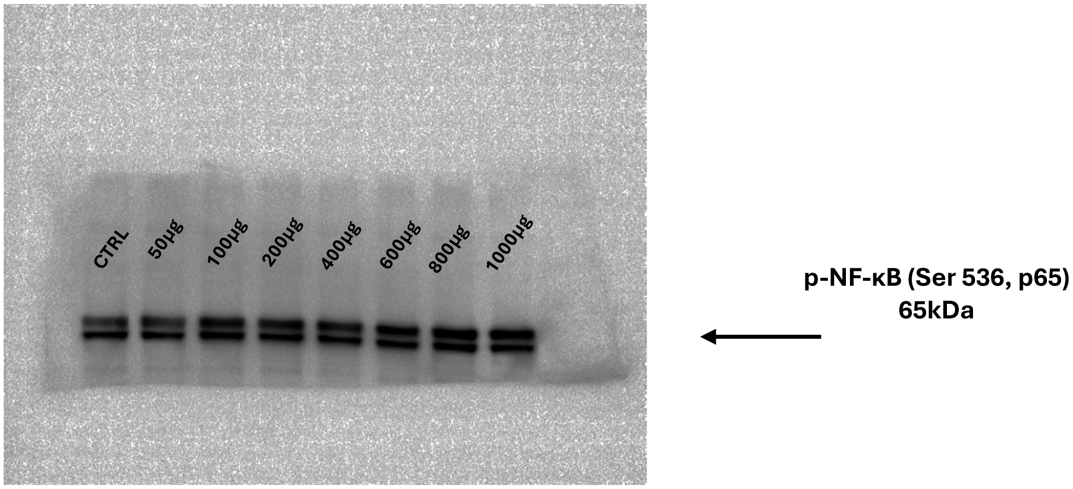

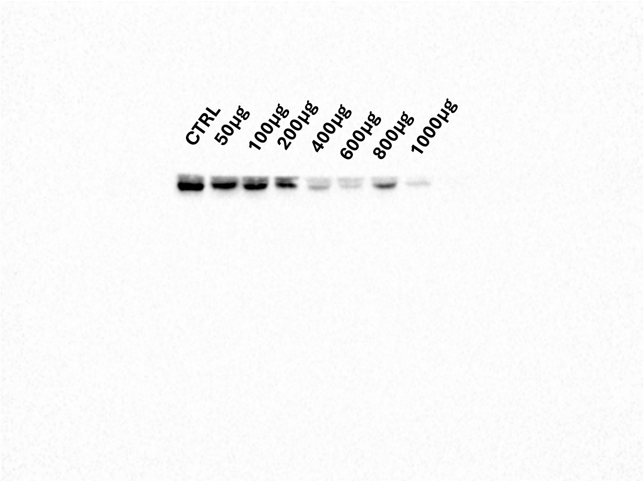

STAT1
84-91kDa

Same photo of STAT1 with contrast adjusted using Bio-Rad Image Lab software:

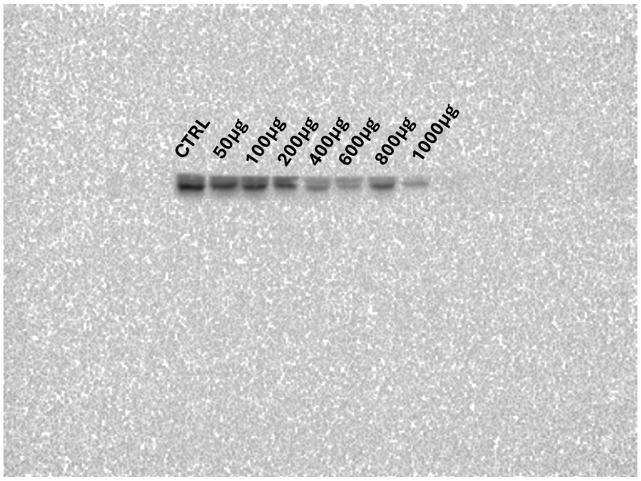

STAT1
84-91kDa

Original representative image of p-STAT1 (Tyr 701) found within the manuscript.

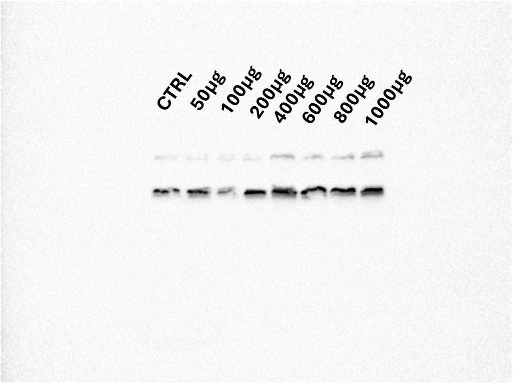

p-STAT1(Tyr 701)
91kDa

Same photo of p-STAT1 (Tyr 701) with contrast adjusted using Bio-Rad Image Lab software:

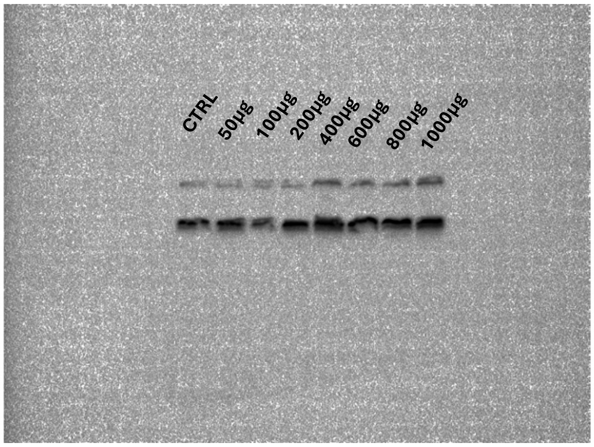

p-STAT1(Tyr 701)
91kDa

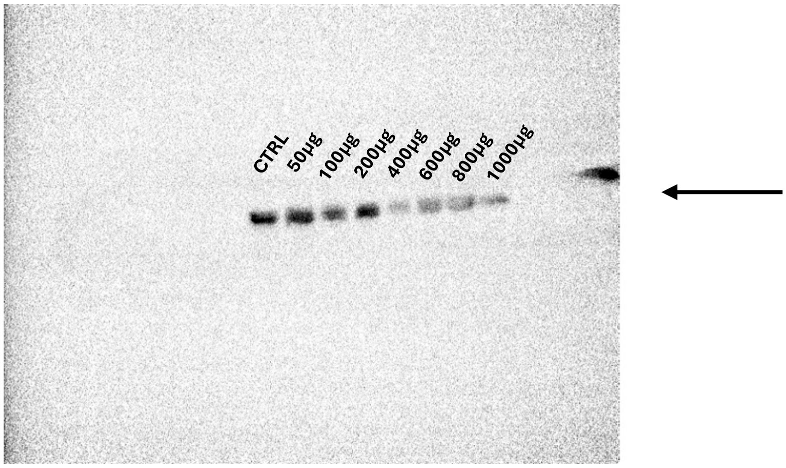

STAT2
97-113kDa

Same photo of STAT2 with contrast adjusted using Bio-Rad Image Lab software:

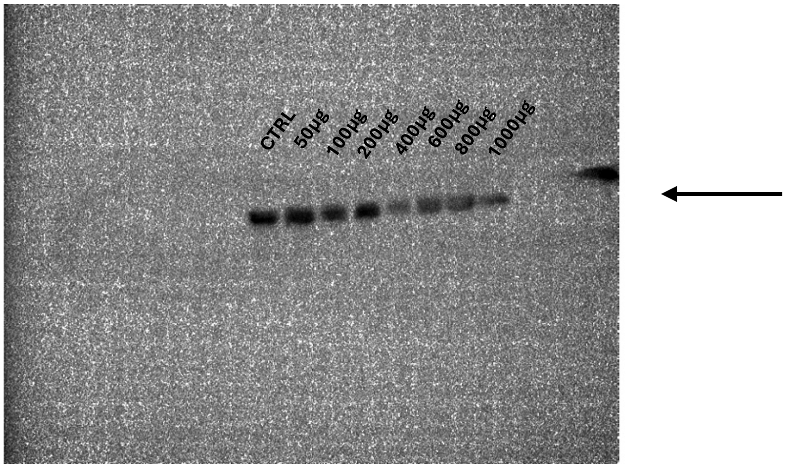

STAT2
97-113kDa

Original representative image of p-STAT2 (Tyr 690) found within the manuscript.

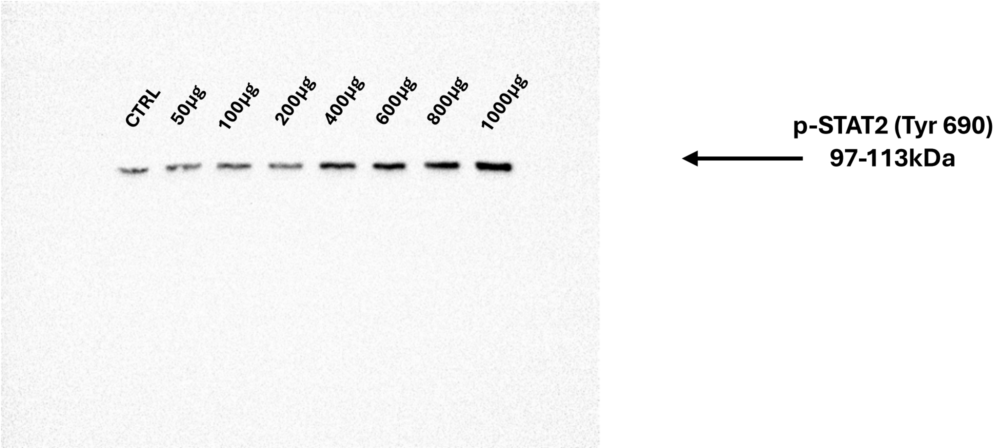

Same photo of p-STAT2 (Tyr 690) with contrast adjusted using Bio-Rad Image Lab software:

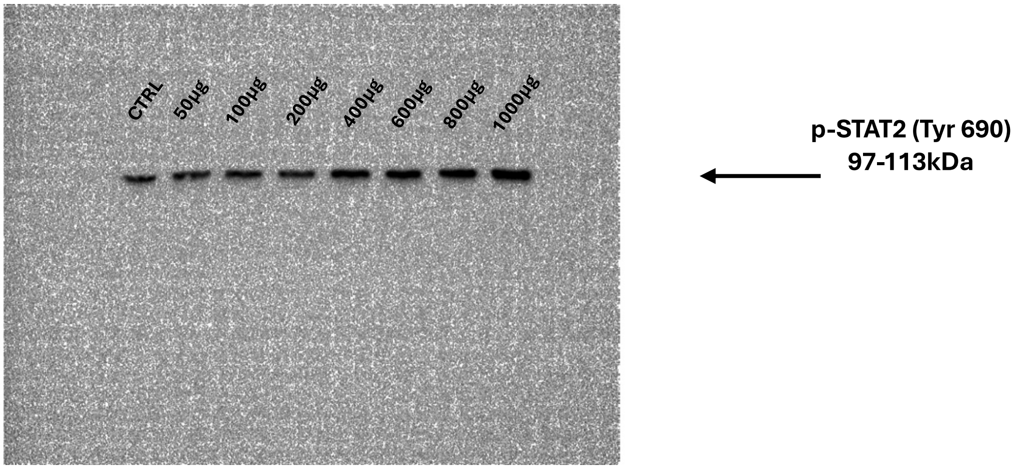

Original representative image of IFN-γ found within the manuscript.

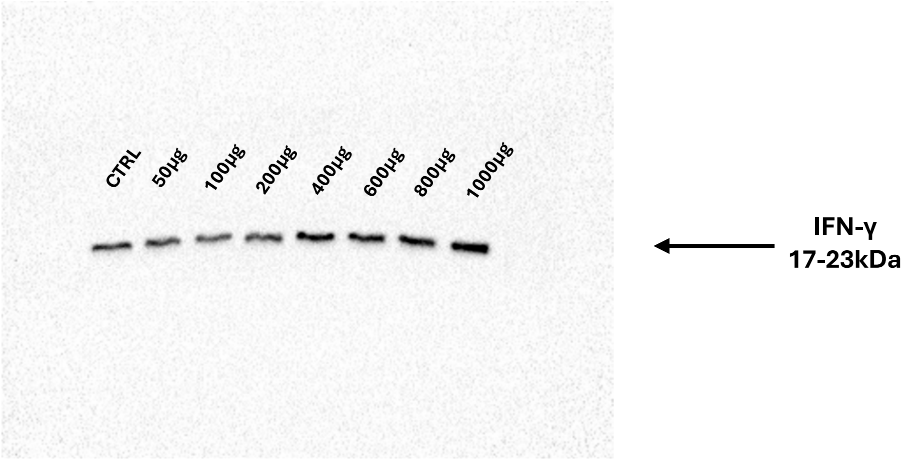

Same photo of IFN-γ with contrast adjusted using Bio-Rad Image Lab software:

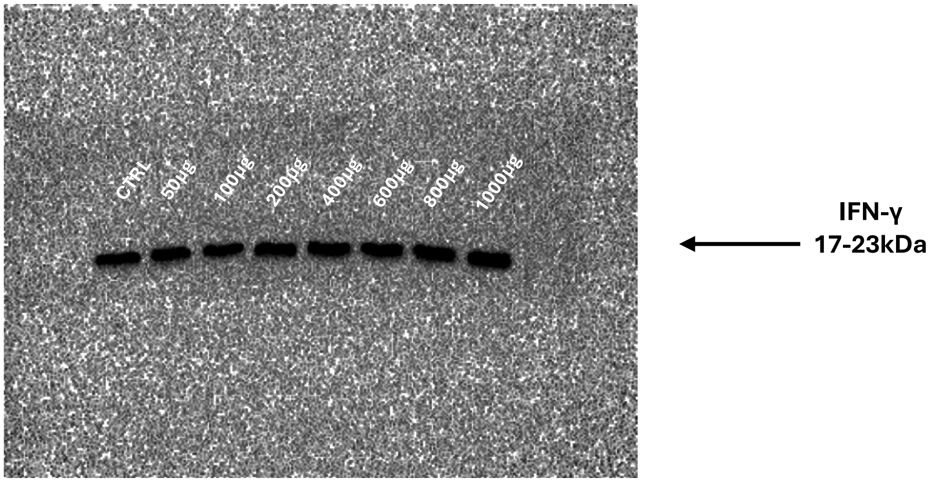

Original representative image of β-actin found within the manuscript.

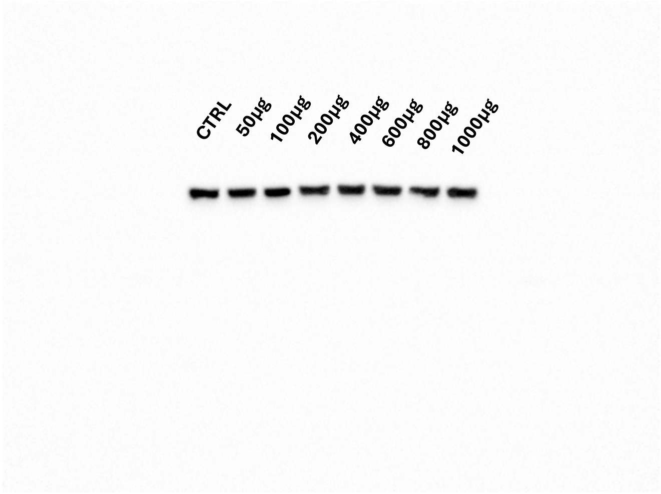

β-Actin (Loading Control)
45kDa

Same photo of β-Actin with contrast adjusted using Bio-Rad Image Lab software:

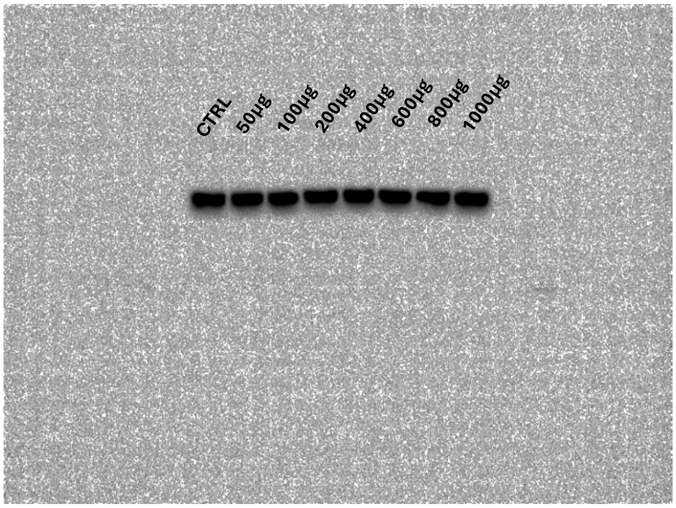

β-Actin (Loading Control)
45kDa

Section 2: Western blot photos found in Figure 2 corresponding for PSP Day 6

Original representative image of TLR4 found within the manuscript.

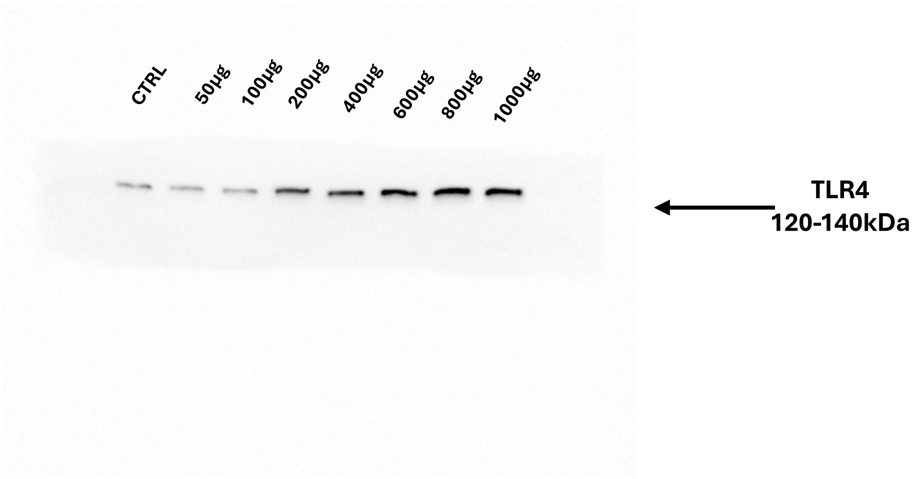

Same photo of TLR4 with contrast adjusted using Bio-Rad Image Lab software:

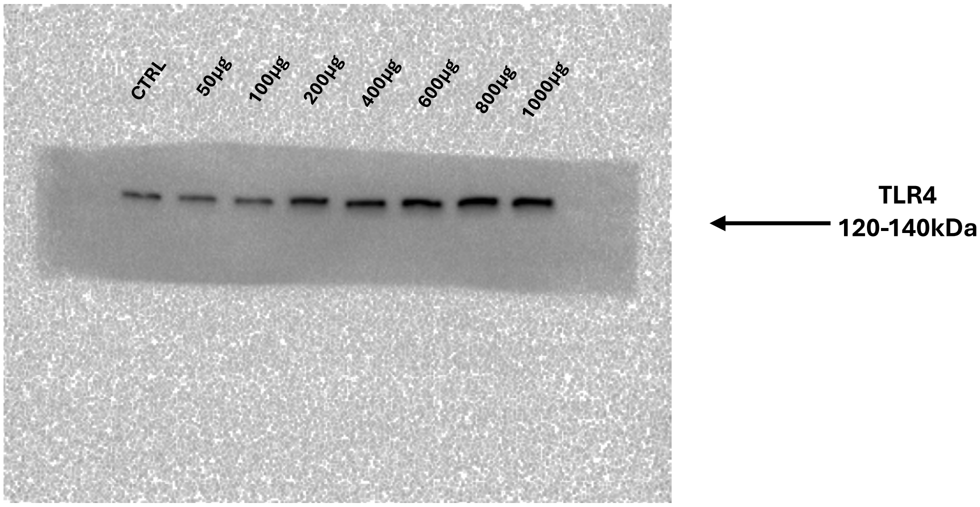

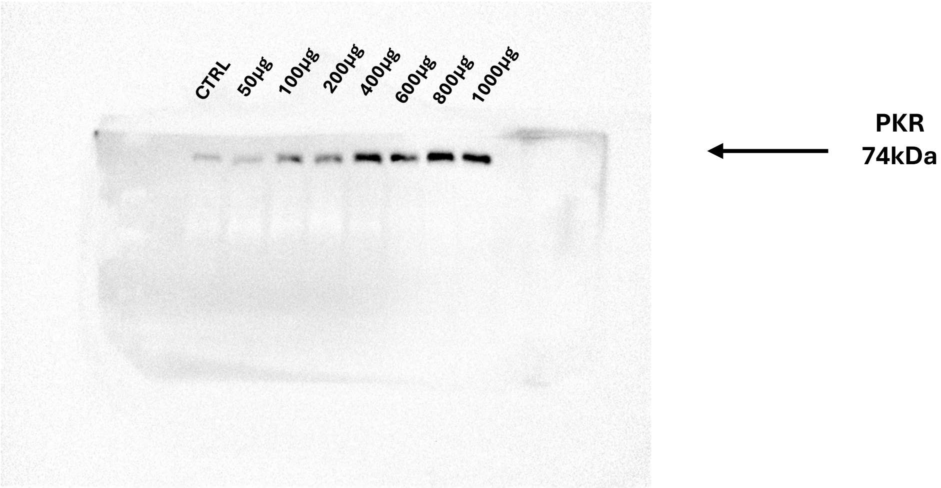

Original representative image of p-PKR (T446) found within the manuscript.

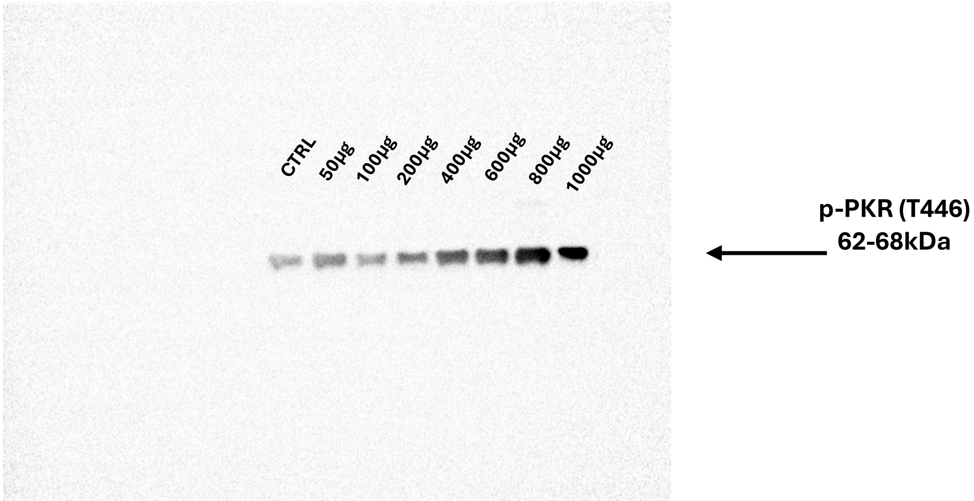

Same photo of p-PKR (T446) with contrast adjusted using Bio-Rad Image Lab software:

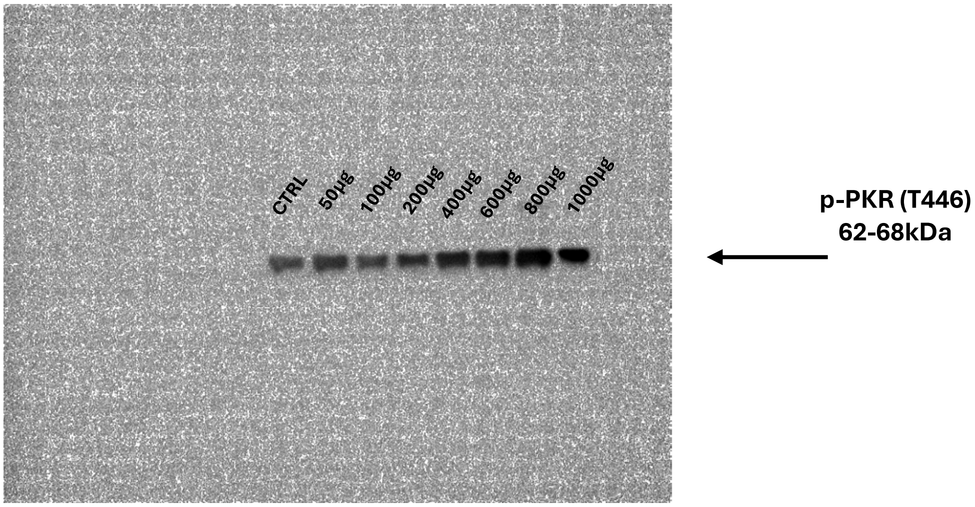

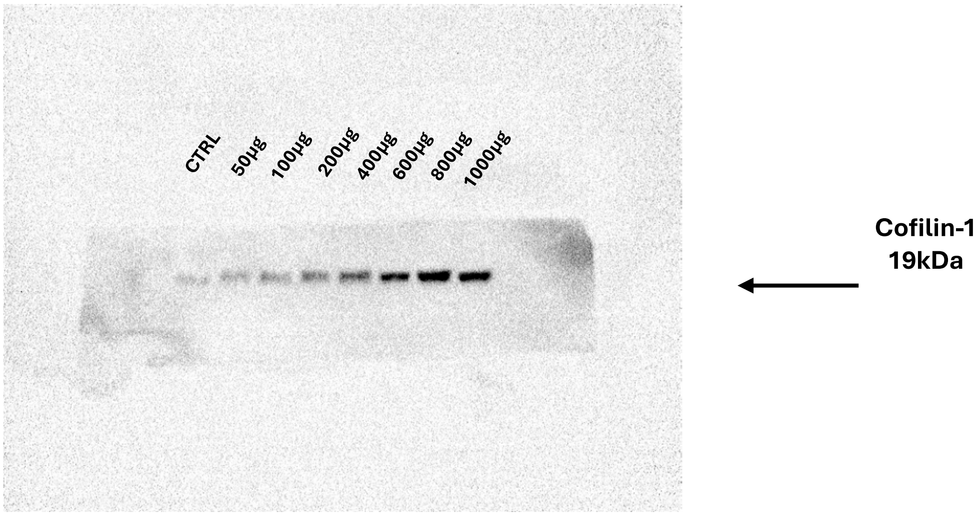

Original representative image of p-Cofilin-1 (Ser3) found within the manuscript.

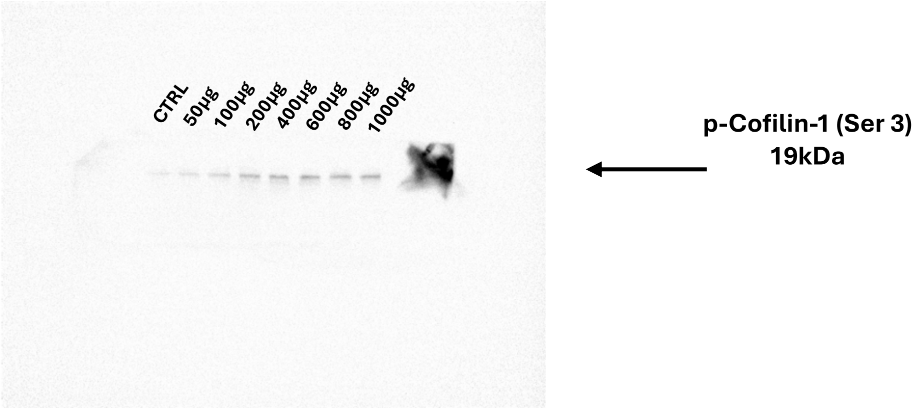

Same photo of p-Cofilin-1 (Ser3) with contrast adjusted using Bio-Rad Image Lab software:

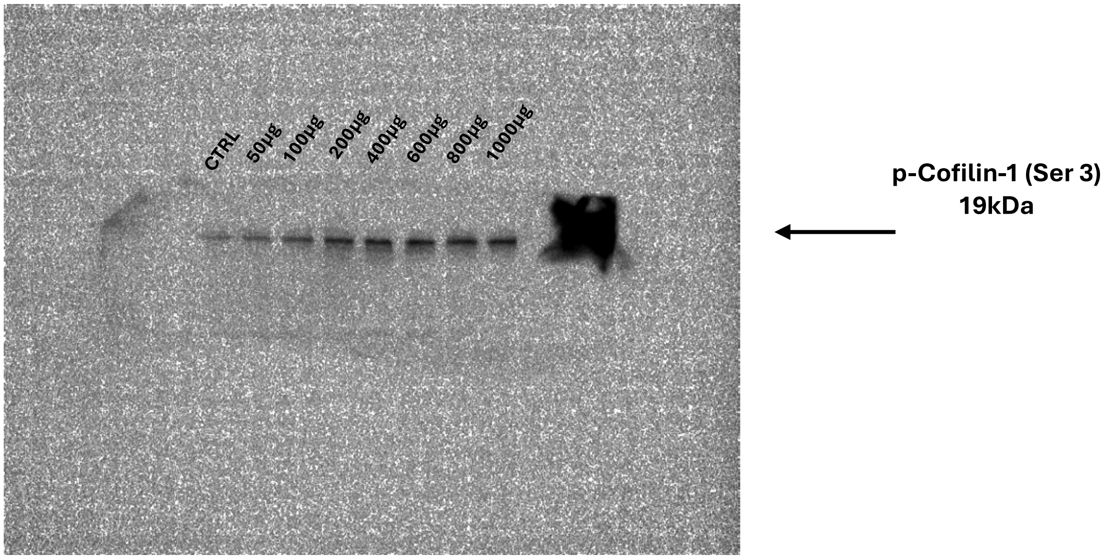

Original representative image of NF-κB found within the manuscript.

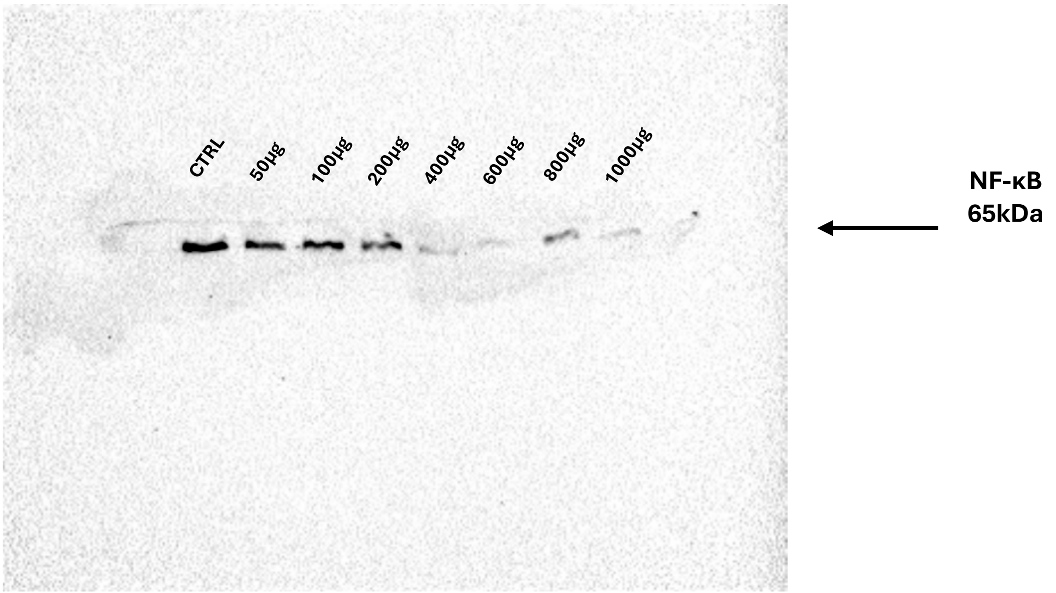

Same photo of NF-κB with contrast adjusted using Bio-Rad Image Lab software:

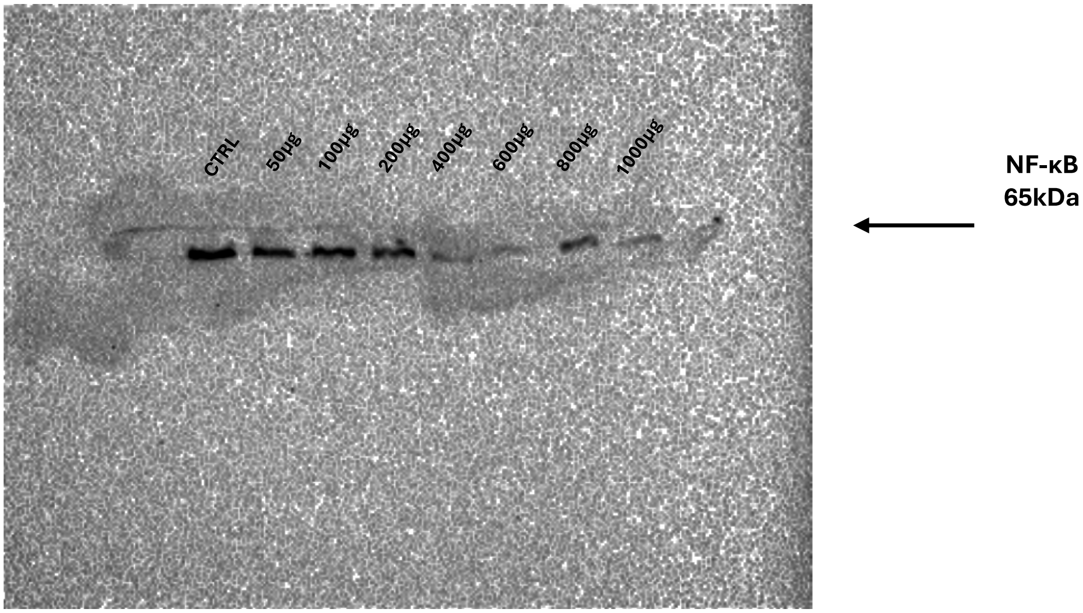

Original representative image of p-NF-κB (Ser536, p65) found within the manuscript.

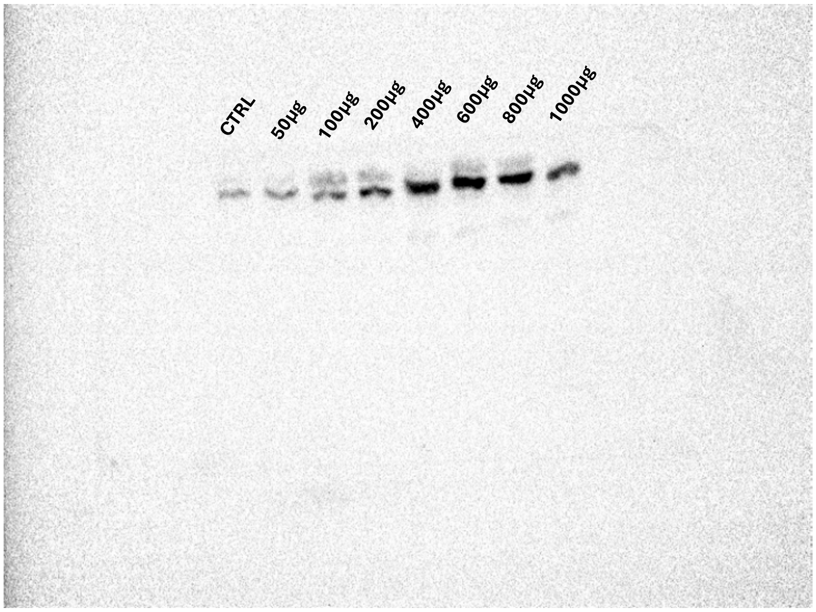

p-NF-κB (Ser 536, p65)
65kDa

Same photo of p-NF-κB (Ser536, p65) with contrast adjusted using Bio-Rad Image Lab software:

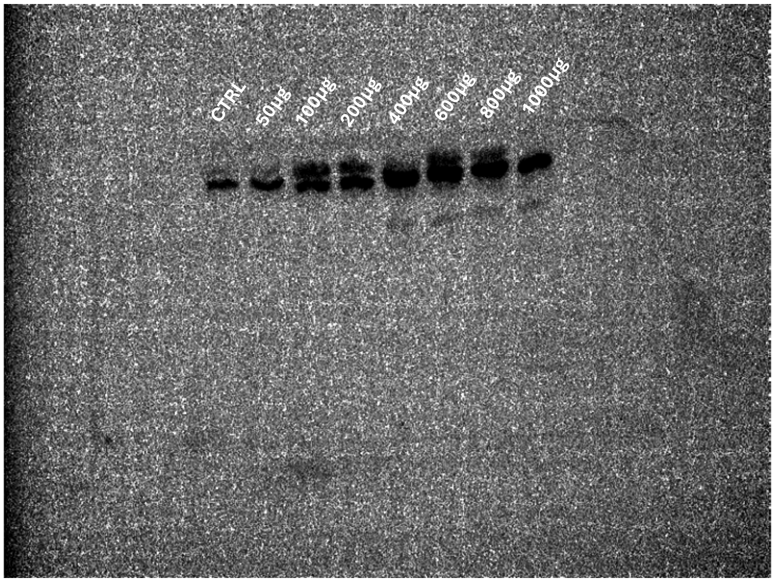

p-NF-κB (Ser 536, p65)
65kDa

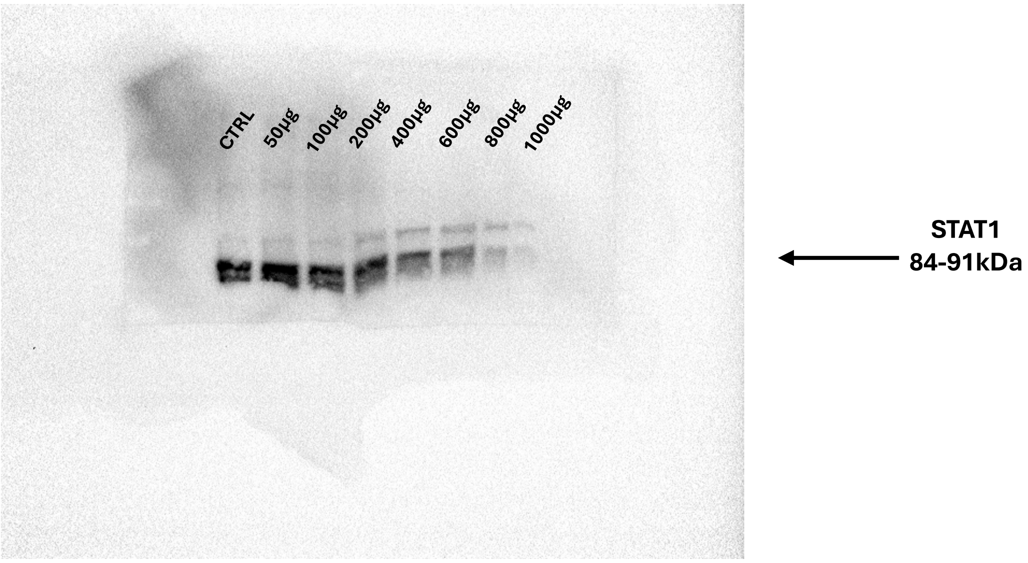

Original representative image of p-STAT1 (Tyr 701) found within the manuscript.

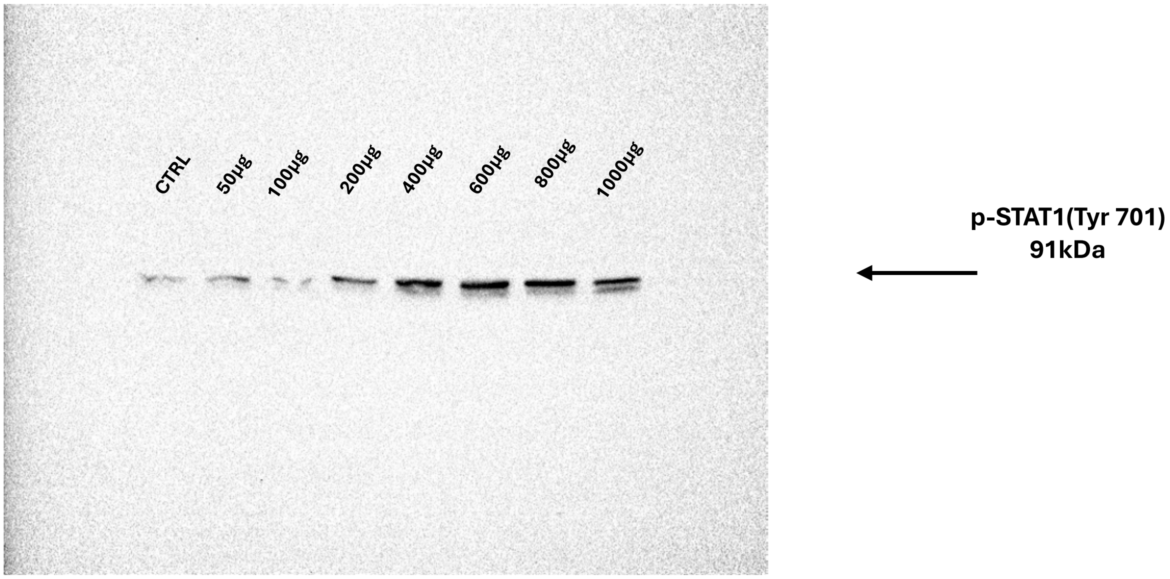

Same photo of p-STAT1 (Tyr 701) with contrast adjusted using Bio-Rad Image Lab software:

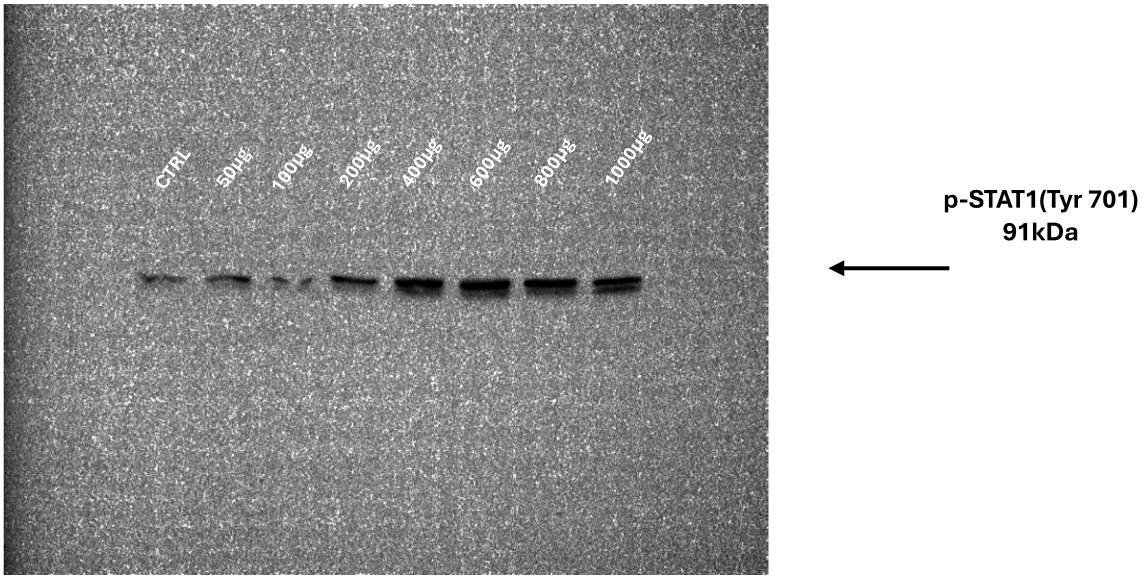

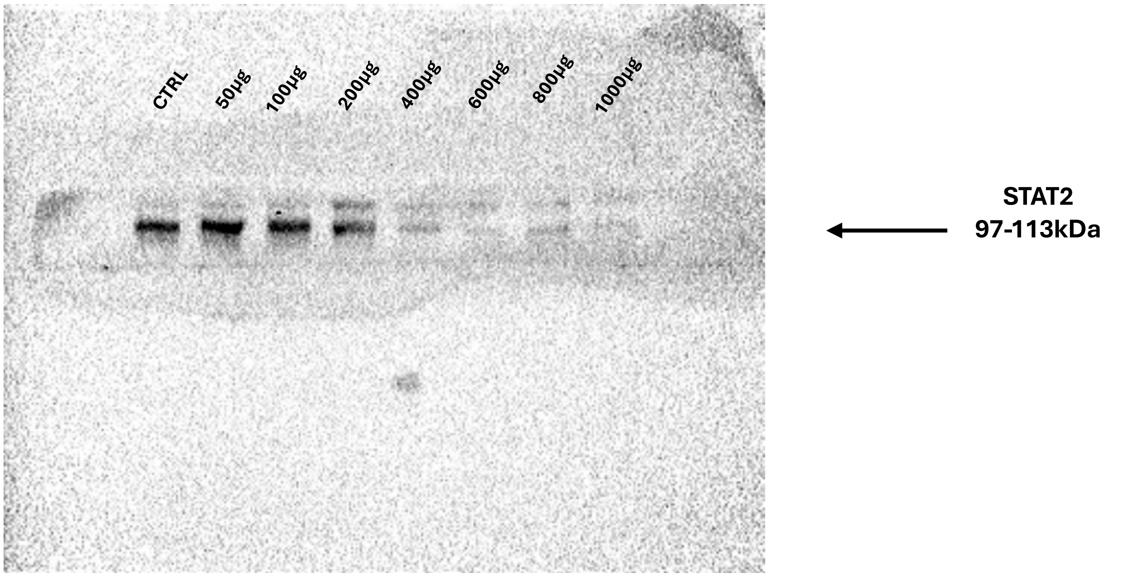

Original representative image of p-STAT2 (Tyr 690) found within the manuscript.

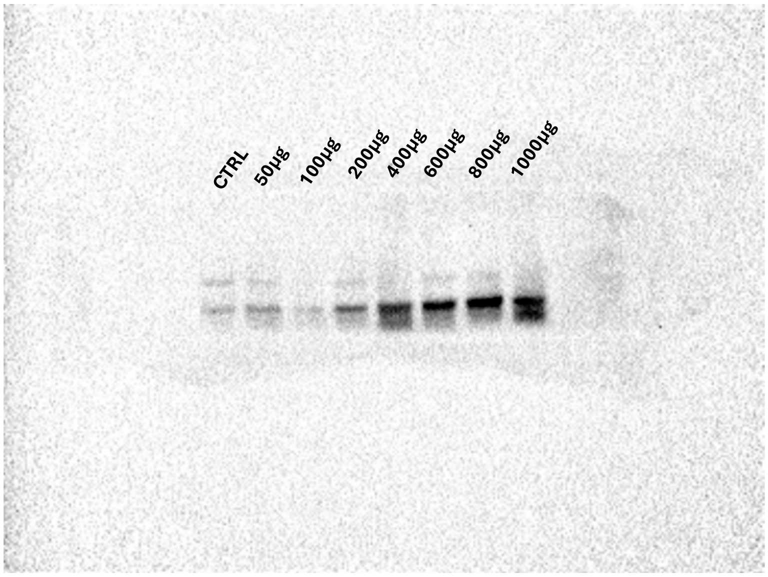

p-STAT2 (Tyr 690)
97-113kDa

Same photo of p-STAT2 (Tyr 690) with contrast adjusted using Bio-Rad Image Lab software:

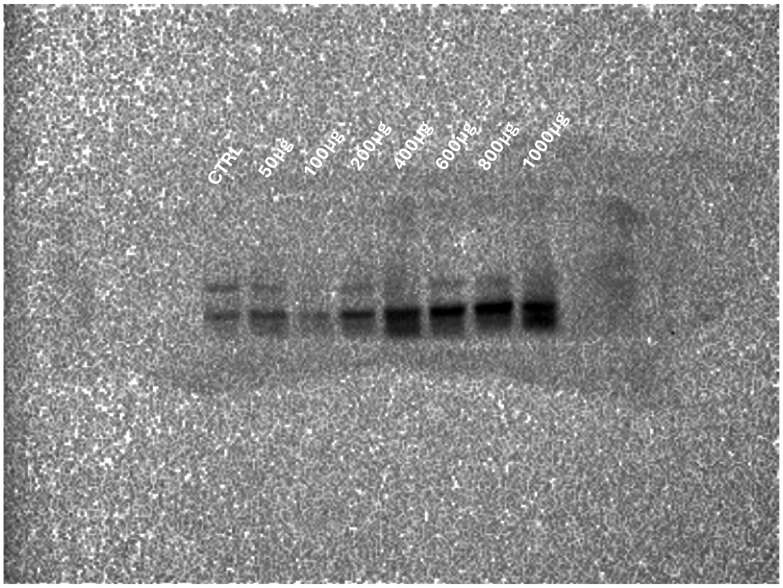

p-STAT2 (Tyr 690)
97-113kDa

X= Unused Lane. Not included in the analysis.

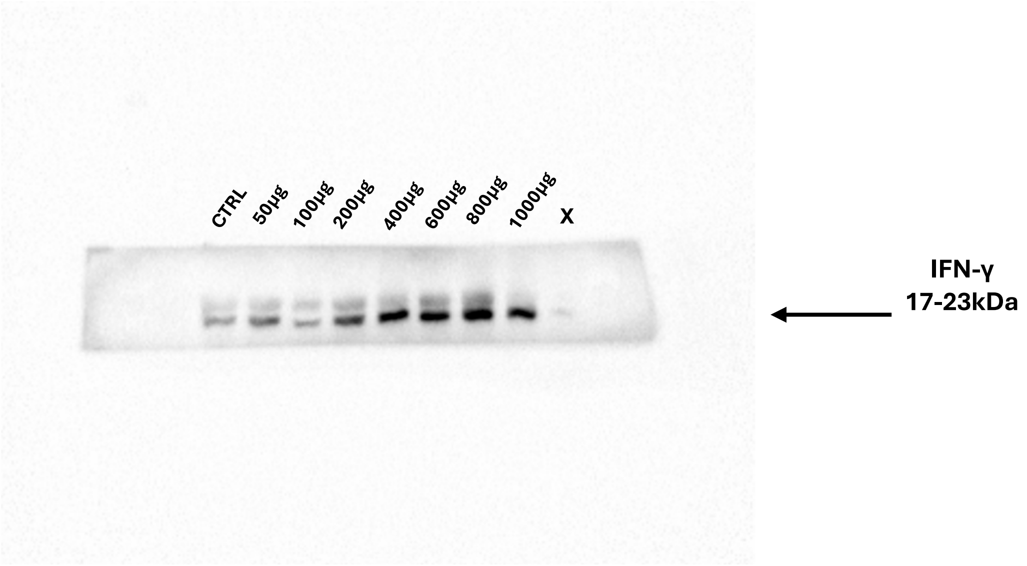

Original representative image of β-actin found within the manuscript.

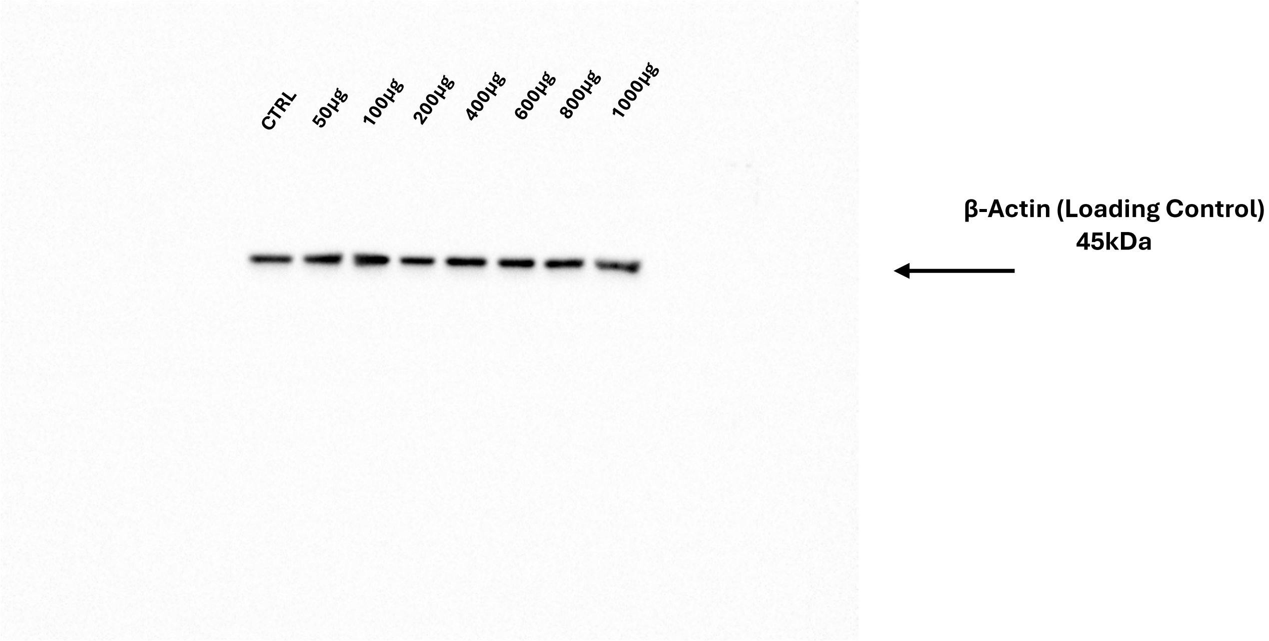

Same photo of β-Actin with contrast adjusted using Bio-Rad Image Lab software:

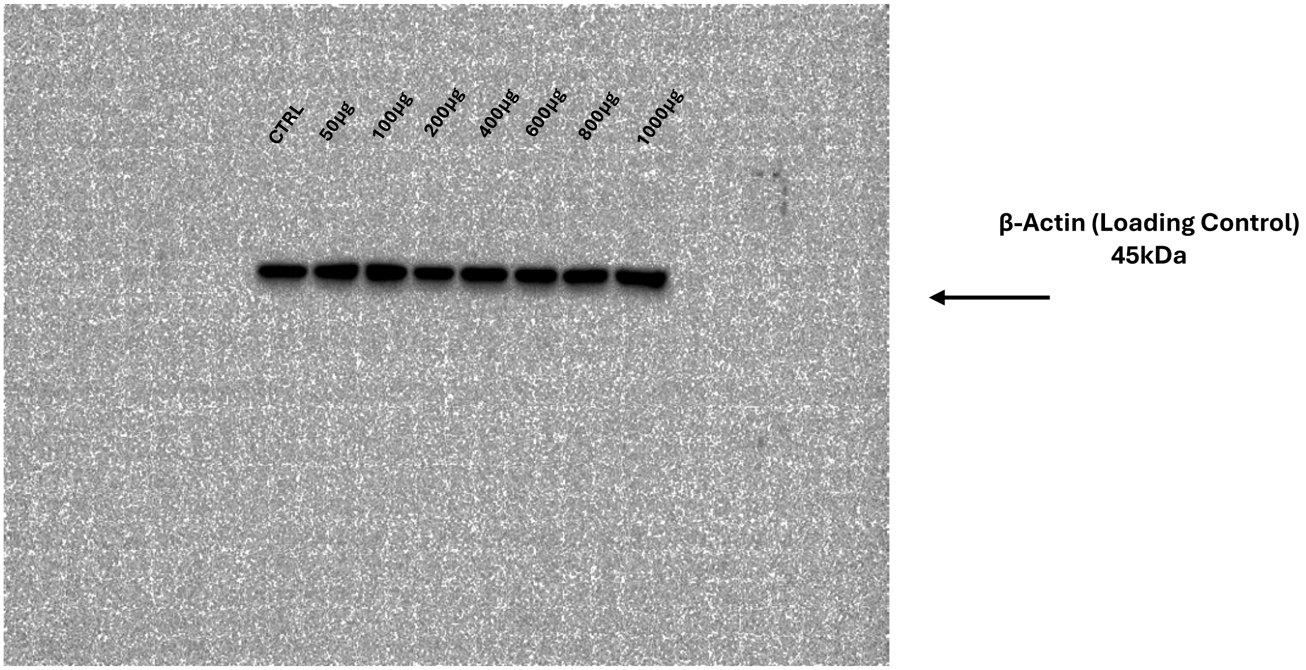

Supplement: Supplementary file 1 [file ijms-27-03661-s001.zip › ijms-4231283-supplementary.pdf]
